# ITS2 metabarcoding analysis complements lichen mycobiome diversity data

**DOI:** 10.1007/s11557-018-1415-4

**Published:** 2018-06-22

**Authors:** Elisa Banchi, David Stankovic, Fernando Fernández-Mendoza, Fabrizia Gionechetti, Alberto Pallavicini, Lucia Muggia

**Affiliations:** 1 Department of Life Sciences, University of Trieste, Via Giorgieri 10, 34127 Trieste, Italy; 2 National Institute of Biology, Marine Biology Station, Fornače 41, 6330 Piran, Slovenia; 3 Institute of Plant Sciences, Karl-Franzens University of Graz, Holteigasse 6, 8010 Graz, Austria

**Keywords:** Ascomycetes, Basidiomycetes, Endophytes, Fungal isolates, Ion torrent, ITS1

## Abstract

Lichen thalli harbor complex fungal communities (mycobiomes) of species with divergent trophic and ecological strategies. The complexity and diversity of lichen mycobiomes are still largely unknown, despite surveys combining culture-based methods and high-throughput sequencing (HTS). The results of such surveys are strongly influenced by the barcode locus chosen, its sensitivity in discriminating taxa, and the depth to which public sequence repositories cover the phylogenetic spectrum of fungi. Here, we use HTS of the internal transcribed spacer 2 (ITS2) to assess the taxonomic composition and diversity of a well-characterized, alpine rock lichen community that includes thalli symptomatically infected by lichenicolous fungi as well as asymptomatic thalli. Taxa belonging to the order Chaetothyriales are the major components of the observed lichen mycobiomes. We predict sequences representative of lichenicolous fungi characterized morphologically and assess their asymptomatic presence in lichen thalli. We demonstrated the limitations of metabarcoding in fungi and show how the estimation of species diversity widely differs when ITS1 or ITS2 are used as barcode, and particularly biases the detection of Basidiomycota. The complementary analysis of both ITS1 and ITS2 loci is therefore required to reliably estimate the diversity of lichen mycobiomes.

**Electronic supplementary material:**

The online version of this article (10.1007/s11557-018-1415-4) contains supplementary material, which is available to authorized users.

## Introduction

The traditional view of lichens as mutualistic, symbiotic associations between one fungus, the mycobiont, and a population of algae, the photobionts (Hawksworth and Honegger 1994), has been reviewed in a more comprehensive and integrative context in which lichens act as microhabitats where multiple fungi (representing diverse classes of Dikarya), algae, and bacteria coexist and likely contribute to the functions of the symbiotic system as a whole (Arnold et al. 2009; Grube et al. 2009; Muggia and Grube 2010; U’Ren et al. 2012; Grube et al. 2015; Spribille et al. 2016; Moya et al. 2017). The high diversity of lichen-associated fungi, and the fact that many species are found in different hosts and habitats, suggested that lichens act as “cradles of symbiotrophic fungal diversification” (Harutyunyan et al. 2008; Arnold et al. 2009; U’Ren et al. 2010, 2012). Recently, the diversity of lichen-associated fungi, hereafter referred to as lichen mycobiomes, has been emphasized by both culture-based methods and high-throughput amplicon sequencing techniques (U’Ren et al. 2010, 2012; Muggia et al. 2016; Fernández-Mendoza et al. 2017).

Multiple ecological guilds of fungi can be found growing associated with lichen thalli (Arnold et al. 2009; Bates et al. 2012; Fernández-Mendoza et al. 2017). One group of lichen-associated taxa is readily recognizable by their phenotypic characters and the conspicuous symptoms of infection shown by their hosts. Such taxa have long been referred to as lichenicolous fungi (Hawksworth 1979, 1981; Lawrey and Diederich 2003, 2016; Hafellner 2015). While the symptomatic occurrence of lichenicolous fungi is restricted to a few lichen hosts, we have recently observed that some lichenicolous fungi are present in other lichens without producing visible symptoms (Fernández-Mendoza et al. 2017). A second component of the lichen mycobiome is formed by species that cannot be detected morphologically, but are widely present within lichen thalli and are abundantly isolated using culture methods (Petrini et al. 1990; Girlanda et al. 1997; Harutyunyan et al. 2008; Muggia et al. 2016). These fungi have been collectively termed endolichenic fungi due to their relatedness to plant endophytes (Arnold et al. 2009); many others are also related to plant pathogens and rock-inhabiting fungi (RIF; Selbmann et al. 2015; Muggia et al. 2016). Finally, a third component is represented by extraneous fungi or fungal propagules found upon or incorporated within lichen thalli without playing any definite ecological role in the lichen community (Fernández-Mendoza et al. 2017). This third component can be derived from other lichen mycobionts present in the community under focus, or from fungi known from different niches. Lichens may act in this way as complex banks of spores and mycelia and would function as suboptimal habitats or reservoirs where the regeneration of local fungal communities can be potentially boosted (Fernández-Mendoza et al. 2017). In this regard, lichen thalli may serve as refuges where such fungi can remain in an immature state until an opportunity arises to occupy more favorable habitats (Muggia et al. 2010; Fernández-Mendoza et al. 2017).

The internal transcribed spacer (ITS) region has been selected as formal DNA barcode for fungi due to its high interspecific variability, conserved primer sites and presence in multiple copies (Blaalid et al. 2013; Schoch et al. 2012). Its length, up to 800 base pairs (bp), is suitable for traditional (Sanger) DNA barcoding, but exceeds the read length required by most second-generation sequencing platforms for DNA metabarcoding, which averages 200–400 bp. For this reason, only one of the two spacers, either ITS1 or ITS2, has been sequenced so far. Even though diversity studies using these new technologies have become more and more common in the last years (Bellemain et al. 2013; Abdelfattah et al. 2015; Miller et al. 2016), it is still debated whether ITS1 or ITS2 has the best taxonomic resolution.

Few studies have dealt with the taxonomic resolution obtained using both the ITS1 and the ITS2 barcodes on the same dataset (Mello et al. 2011; Blaalid et al. 2013; Bazzicalupo et al. 2013; Monard et al. 2013; Orgiazzi et al. 2013). They have been carried out on both Ascomycota (Nilsson et al. 2009; Bellemain et al. 2013) and Basidiomycota (Badotti et al. 2017). Taxonomic bias can be introduced by the choice of primers, as these cause a higher number of mismatches in different taxa (Bellemain et al. 2013; Tedersoo et al. 2015; Tedersoo and Lindahl 2016). Some studies also reported that the two spacers are prone to preferential amplification at different levels (Nilsson et al. 2009; Mello et al. 2011; Bellemain et al. 2013; Bazzicalupo et al. 2013; Monard et al. 2013). Basidiomycetes have on average longer amplicon sequences for the ITS2, and since the shorter sequences are preferentially sequenced with high-throughput sequencing (HTS), the use of ITS2 would favor the detection of ascomycetes (Bellemain et al. 2013). On the other hand, ITS1 often contains an intron that extends its sequence at the 5′-end (Martin and Rygiewicz 2015), thereby promoting an over-representation of those sequences that lack the intron (Bazzicalupo et al. 2013). Because ITS2 is more frequently represented in public databases, has a higher number of available sequences, and offers a better taxonomic resolution, it has been proposed as the better choice for parallel sequencing (Nilsson et al. 2009). In some cases, however, no substantial differences between ITS1 and ITS2 were recovered (Blaalid et al. 2013; Badotti et al. 2017). Finally, there are numerous studies that consider a single spacer, either the ITS1 or ITS2 (Bellemain et al. 2013; Langarica-Fuentes et al. 2014; U'Ren et al. 2014; Miller et al. 2016; Fernández-Mendoza et al. 2017).

As fungal metabarcoding studies have used different HTS platforms (see Cuadros-Orellana et al. 2013 for a review), different bioinformatic pipelines have been proposed (White et al. 2013; Bálint et al. 2014; Gweon et al. 2015). These have been developed based on experience gained from data analyses of prokaryote datasets (Hibbett 2016). However, no standard procedure has been established so far for fungal sequence data. The analyses seem strongly dependent on the working hypotheses of each study and on the type of sequence at hand. As the majority of studies target fungal communities to uncover unknown diversity, an important and ongoing problem is the definition of those sequences lacking an assigned taxonomy (Nilsson et al. 2016). For this reason, many sequences still remain identified as “uncultured fungus.” In addition, many fungi have not yet been sequenced and cannot offer reference sequences for ongoing studies (Hibbett 2016). Both cases emerge as main issues in investigations of lichen mycobiome(s) where unidentified fungi represent the largest proportion.

Previous studies processed high-throughput amplicon sequencing data from lichens, considering thalli of different growth forms and others infected by symptomatic lichenicolous fungi (Bates et al. 2012; U’Ren et al. 2012, 2014; Fernández-Mendoza et al. 2017). These studies demonstrated that lichens and their mycobiomes are suitable subjects for implementing bioinformatic analyses of fungal metabarcoding. Nonetheless, individual environmental specimens have rarely been used for the characterization of fungal assemblages (Yahr et al. 2016); this approach was initiated only recently by Fernández-Mendoza et al. (2017). The authors highlighted the suitability of single lichen thalli for reliable estimation of the mycobiome diversity within. Fernández-Mendoza et al. (2017) studied the mycobiome diversity by applying 454 pyrosequencing to a well-characterized set of lichens (Fleischhacker et al. 2015; Muggia et al. 2016), comparing thalli visibly infected by lichenicolous fungi to others devoid of detectable infections. The authors expected fungal diversity within the lichen community and sought to determine whether lichenicolous fungi were asymptomatically present in typical and atypical lichen hosts, and whether the presence of symptomatic lichenicolous fungi correlated with the diversity of the other intrathalline, asymptomatically occurring fungi. They also attempted to gauge the potential specificity of thallus mycobiomes among different lichen hosts (Fernández-Mendoza et al. 2017).

**Fig. 1:**
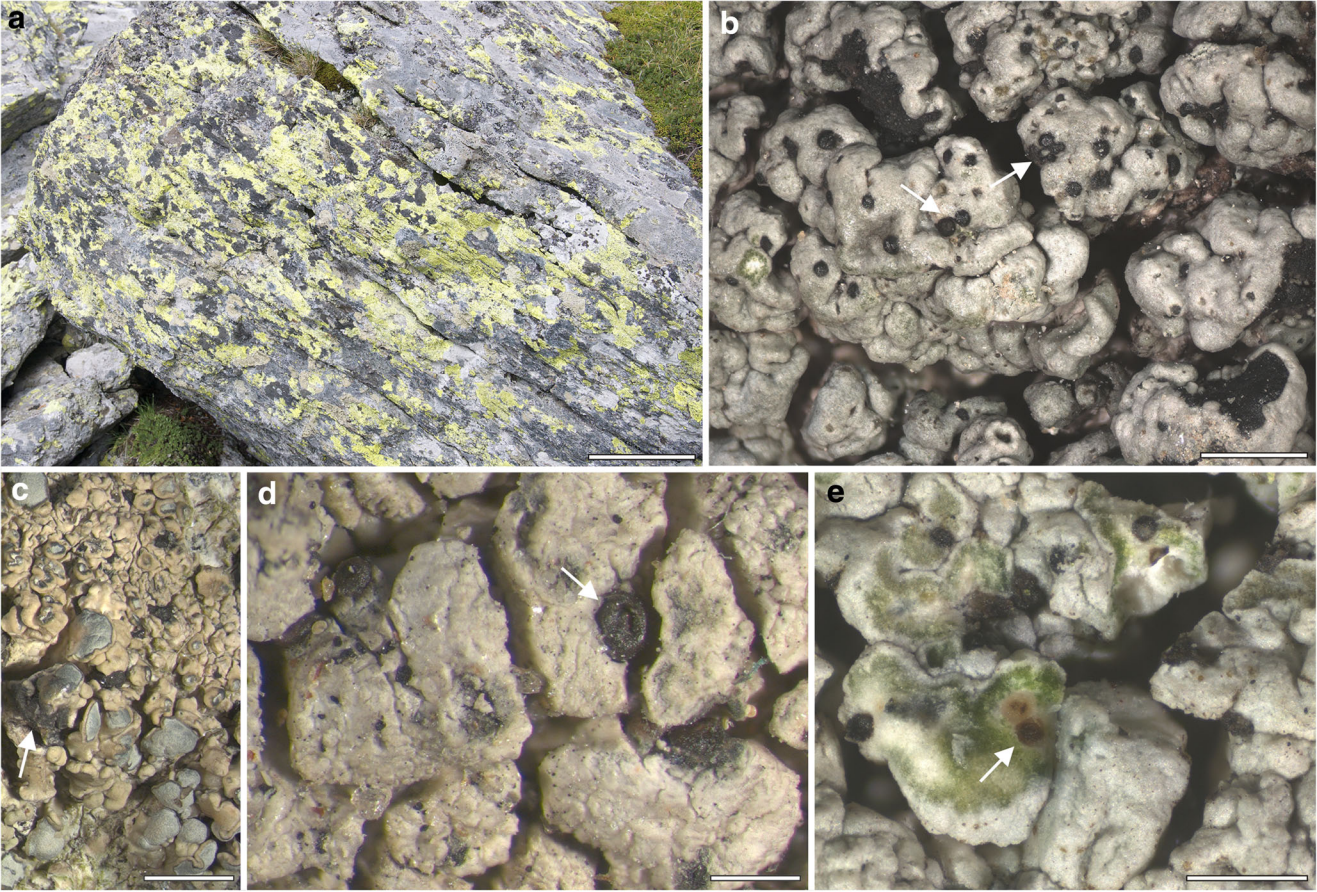
**a** Alpine community of rock-inhabiting lichens on siliceous boulders. **b**–**e** Symptomatically infecting lichenicolous fungi on lichen host thalli: **b** *Muellerella atricola* on *Tephromela atra*, **c** *Sphaerellothecium atrynae* on *Lecanora swartzii*, **d** *Sagediopsis fissurisedens* on *Aspilidea myrinii*. Arrows indicate the recognizable, phenotypic characters of the lichenicolous fungi: **b**, **d** perithecia immerse at the margins of thallus areoles, **c** dark, melanized discoloration in which perithecia are present, **e** conidiomata (pycnidia) containing conidiospores immerse in the thallus areoles. Scale bars: **a** = 15 cm; **b** = 1 mm; **c** = 2 mm; **d**, **e** = 0.5 mm

As studies of lichen mycobiomes may fail to recover the complete taxonomic profile when using either the ITS1 or ITS2 regions individually, both regions should be examined to obtain more accurate estimates of species diversity. Here, we re-evaluate the fungal communities (Fig. 1) studied by Fernández-Mendoza et al. (2017) by sequencing the ITS2 fragment using the Ion Torrent (Thermo Fisher Scientific) platform. We assess the taxon diversity detected with the ITS2 barcode, focusing on new fungal sequences potentially corresponding to lichenicolous fungi, and compare the new ITS2 dataset with the previously analyzed ITS1 results. Because Fernández-Mendoza et al. (2017) used the 454 pyrosequencing method, we also evaluate the performance of high-throughput amplicon sequencing approaches in the analyses of lichen mycobiomes.

## Material and methods

### Sampling

Lichen samples and their DNA extractions correspond to those recently analyzed by Fernández-Mendoza et al. (2017) and their preparation corresponds to that reported in Fernández-Mendoza et al. (2017). The samples are part of a comprehensive study on fungi associated with lichens in alpine rock communities which has been conducted for the past 4 years (Fleischhacker et al. 2015; Muggia et al. 2016, 2017; Fernández-Mendoza et al. 2017). Samples (Table 1) were collected in ten plots (each about 300 m^2^) at an altitude of 1800–1900 m on the Koralpe mountain range in Eastern Austria as reported in Fleischhacker et al. (2015). DNA was extracted from 26 samples, including 25 crustose and one foliose lichens, using the DNeasy Plant Mini Kit (Qiagen, Austria). Crustose lichens were predominant in the selected community; thalli consisted of contiguous areoles tightly adhering to the substrate. The single foliose thallus was represented by *Umbilicaria cylindrica*, which is attached to the substrate by a central holdfast (umbilicus). Half of the samples (13) were symptomatically infected by lichenicolous fungi, including teleomorphic and anamorphic ascomycetes (Fig. 1 and Table 1). This means that lichenicolous fungi could be observed on the thallus and identified using light microscopy. The other 13 thalli were devoid of symptomatic fungal infections; we refer to them as “asymptomatic samples,” without ruling out the cryptic presence of lichenicolous fungi within the thalli. The dataset includes a total of 10 species of symptomatic lichenicolous fungi and 13 species of lichen hosts (Table 1).

**Table 1:** Summary of dataset description and sequencing results. The table includes sample numbers, name of the lichen host and of the symptomatic lichenicolous fungus, the total number of reads (Tot), the number of reads that passed quality filter (Qf), and the number of reads (ITS2 extracted and chimera filtered) of the three datasets analyzed (complete, “no host,” “no myco”). The number of observed OTUs in each sample is given for the entire sample, whereas values of Chao1 and Shannon diversity indexes are calculated on datasets rarefied to 7133 for the complete, to 1073 for the “no host,” and to 1060 reads for the “no myco.” The diversity indexes are not reported where sequencing depth was not sufficient (/)

			Reads					OTUs			Chao1 estimate			Shannon diversity index		
Sample	Lichen host	Infecting fungus	Tot	Qf	Complete	“No host”	“No myco”	Complete	“No host”	“No myco”	Complete	“No host”	“No myco”	Complete	“No host”	“No myco”
A032	*Umbilicaria cylindrica*	–	16,964	13,049	11,898	217	166	111	35	19	111 ± 24	/	/	2.19 ± 0.05	/	/
A138	*Candelariella vitellina*	–	16,544	12,822	12,285	1348	1213	372	172	131	374 ± 57	185 ± 32	140 ± 24	3.6 ± 0.09	4.06 ± 0.67	3.57 ± 0.6
A172	*Rhizocarpon geographicum*	–	20,395	14,775	14,329	1901	1898	221	175	172	215 ± 44	177 ± 41	172 ± 36	2.67 ± 0.07	4.85 ± 0.72	4.85 ± 0.7
A194	*Rhizocarpon geographicum*	*Endococcus macrosporus*	18,682	15,256	14,643	1073	1060	229	117	109	224 ± 44	120 ± 23	116 ± 20	1.71 ± 0.07	3.97 ± 0.56	3.9 ± 0.55
A227	*Lecanora swartzii*	–	16,328	13,409	12,360	86	54	549	22	9	568 ± 75	/	/	3.32 ± 0.1	/	/
A229	*Psorinia conglomerata*	–	21,095	15,997	14,283	3453	160	417	332	64	414 ± 107	299 ± 99	/	2.99 ± 0.07	4.21 ± 0.57	/
A243	*Lecanora polytropa*	–	16,726	13,489	13,176	224	40	135	64	18	137 ± 29	/	/	0.58 ± 0.04	/	/
A280	*Tephromela atra*	*Skyttea tephromelarum*	11,074	7805	7140	863	854	201	90	87	242 ± 54	/	/	2.6 ± 0.05	/	/
A360	*Lecanora intricata*	–	18,643	13,619	12,329	5671	5326	179	127	77	183 ± 41	90 ± 38	57 ± 37	1.89 ± 0.06	1.29 ± 0.27	0.83 ± 0.17
A361	*Tephromela atra*	–	13,883	11,180	11,040	44	43	141	26	25	161 ± 49	/	/	1.14 ± 0.03	/	/
A368	*Lecanora bicincta*	–	14,797	12,120	11,836	36	4	220	17	2	223 ± 54	/	/	1.52 ± 0.07	/	/
A405	*Rhizocarpon geographicum*	*Muellerella pygmaea-Rg*	18,630	11,455	10,963	1377	1363	265	232	228	267 ± 40	235 ± 31	238 ± 26	1.96 ± 0.08	6.16 ± 1.02	6.14 ± 1.02
A418	*Lecanora polytropa*	*Lichenoconium lecanorae*	17,149	13,200	12,196	588	305	208	147	80	215 ± 53	/	/	2.75 ± 0.05	/	/
A420	*Aspilidea myrinii*	–	13,490	8955	8147	593	435	295	61	51	313 ± 45	/	/	3.43 ± 0.06	/	/
A434	*Lecanora polytropa*	*Lichenoconium lecanorae*	16,335	12,172	11,296	7370	7223	557	526	491	579 ± 73	426 ± 148	400 ± 158	5.06 ± 0.11	4.53 ± 0.59	4.46 ± 0.62
A440	*Tephromela atra*	*Muellerella atricola*	14,501	11,034	9276	1371	1321	215	129	114	244 ± 60	139 ± 35	128 ± 31	2.06 ± 0.07	4.14 ± 0.52	3.95 ± 0.49
A476	*Psorinia conglomerata*	–	16,627	13,164	12,803	2939	655	417	352	132	456 ± 117	325 ± 98	/	2.98 ± 0.09	4.64 ± 0.7	/
A482	*Lecanora polytropa*	*Cercidospora epipolytropa*	11,479	9472	9171	471	446	166	79	67	168 ± 28	/	/	1.28 ± 0.06	/	/
A608	*Aspilidea myrinii*	*Sagediopsis fissurisedens*	16,780	13,002	12,326	7964	7858	362	158	146	370 ± 71	105 ± 51	105 ± 60	2.52 ± 0.07	0.76 ± 0.19	0.65 ± 0.2
A622	*Varicellaria lactea*	*Stigmidium eucline*	25,805	12,400	12,068	5296	4822	326	204	143	323 ± 61	149 ± 64	107 ± 45	3.84 ± 0.06	2.57 ± 0.37	2.06 ± 0.33
A623	*Acarospora fuscata*	–	19,532	16,069	15,939	38	32	96	19	13	97 ± 34	/	/	1.1 ± 0.03	/	/
A636	*Lecidea lapicida*	*Muellerella pygmaea s.s.*	3499	496	454	230	230	52	37	37	/	/	/	/	/	/
A670	*Lecanora polytropa*	*Muellerella pygmaea-Lp*	12,531	9404	8923	6447	6192	360	308	277	361 ± 49	247 ± 92	228 ± 81	3.41 ± 0.1	2.33 ± 0.47	2.1 ± 0.4
A792	*Lecidea lapicida*	–	17,241	13,683	12,326	863	79	184	128	27	183 ± 38	/	/	1.38 ± 0.06	/	/
A809	*Tephromela atra*	*Taeniolella atricerebrina*	24,994	20,106	19,353	7980	7964	304	198	190	290 ± 81	181 ± 114	143 ± 68	1.99 ± 0.06	0.94 ± 0.22	0.91 ± 0.25
A832	*Lecanora bicincta*	*Arthonia varians*	9016	7403	7133	85	68	164	23	9	216 ± 54	/	/	1.11 ± 0.05	/	/
Symptomatically infected samples			200,475	143,205	134,942	41,115	39,706	3409	2248	1978	244 ± 95	238 ± 125	215 ± 118	2.56 ± 1.10	3.46 ± 1.88	3.29 ± 1.93
Uninfected samples			222,265	172,331	162,751	17,413	10,105	3337	1530	740	216 ± 115	259 ± 120	133 ± 43	2.25 ± 0.99	4.14 ± 1.40	3.34 ± 1.84
Total			422,740	315,536	297,693	58,528	49,811	6746	3778	2718	229 ± 107	246 ± 123	193 ± 110	2.40 ± 1.06	3.72 ± 1.74	3.31 ± 1.91

### Molecular analysis and sequencing

The fungal nuclear ribosomal ITS2 region was amplified with the forward primer ITS3 and the reverse primer ITS4 (White et al. 1990). The amplicons for HTS were obtained by performing two PCR amplifications. The first PCR amplification used the forward and the reverse primers ITS3 and ITS4 modified with GC rich universal tails on the 5′-end (Carew et al. 2013). The 5′-end tail was identical to the tail applied on the 3′-end of the barcodes used in the second PCR. The first PCR reaction mix contained 3 μl DNA template (10–20 ng), 3 μl HotMasterMix (5PRIME, Fisher Scientific), 0.5 μl BSA 10× (Sigma-Aldrich), 0.75 μl EvaGreen™ 20× (Biotium), 0.5 μl forward primer ITS3 (10 μM), and 0.5 μl reverse primer ITS4 (10 μM) in a final volume of 15 μl. The PCR amplifications were performed with CFX 96™ PCR System (Bio-Rad) with the following cycling profile: 94 °C for 2 min and 30 cycles at 94 °C for 20 s, 55 °C for 20 s, 65 °C for 40 s followed by a final extension at 65 °C for 2 min. A negative control was used to verify the absence of non-specific amplification products along the whole amplification and sequencing process.

The second PCR amplification (switch PCR) was required for the multiplex sequencing and the attachment of the barcodes. It used primers modified with an “A” adaptor and a sample-specific 10-bp barcode to the 5′-end of the forward primer, and a P1 adaptor to the 5′-end of the reverse primer. The reaction was performed in a mix containing 5 μl of the first PCR product, 20 μl HotMasterMix (5PRIME), 2.5 μl EvaGreen™ 20× (Biotium), 1.5 μl forward primer (10 μM), and 1.5 μl reverse primer (10 μM) in a final volume of 50 μl. PCR conditions were the same as for the first PCR amplification but were repeated for 8 cycles. All the amplicons were checked for their quality and size by agarose gel electrophoresis and normalized using Mag-Bind® Normalizer Kit (Omega Biotek). Product concentrations were checked with NanoDrop® 2000 (Thermo Fisher Scientific). The amplicons of the different samples were pooled together in equimolar amounts and the resulting barcoded library was measured with Qubit™ Fluorimeter (Thermo Fisher Scientific) and sequenced with an in-house Ion Torrent Personal Genome Machine™ (PGM, Thermo Fisher Scientific) using a 314™ chip (Thermo Fisher Scientific) for a maximum read length of 400 bp.

### Data analysis

**Fig. 2:**
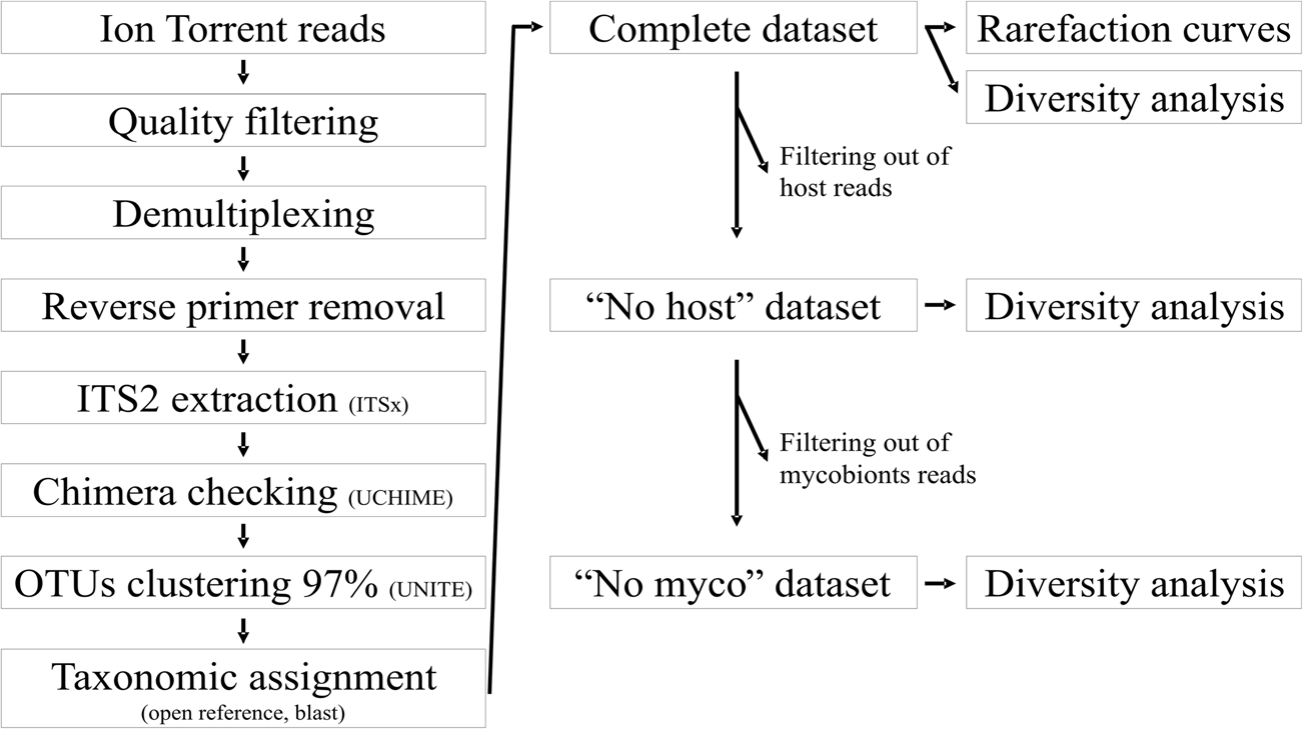
Flowchart of the analytical pipeline implemented in QIIME and performed for the analyses of the fungal ITS2 dataset. The programs used are reported in parentheses

QIIME v.1.8.1 (Caporaso et al. 2010) was used to process the sequence data (Fig. 2). High-quality sequences were demultiplexed (minimum length 150 bp, maximum length of homopolymer 8, maximum number of primer mismatches 3). Minimum average quality score was set to 20 (Kemler et al. 2013; Tang et al. 2015). Reverse primers and barcodes were removed, and reads that did not pass through the filtering were discarded. In order to retain only fungal reads, the ITS2 region was extracted with ITSx v.1.0.11 (Bengtsson-Palme et al. 2013) selecting the fungal (F) profile option. Chimeric reads were identified and filtered out with UCHIME v.4.0 algorithm using the reference dataset updated on 1 January 2016 (Edgar et al. 2011; Nilsson et al. 2015) to obtain the final, high-quality dataset, here referred as complete dataset. Operational taxonomic units (OTUs) were picked at 97% similarity with open-reference strategy and UNITE database, updated on November 2016 (Kõljalg et al. 2013). OTU taxonomy was assigned using NCBI public nucleotide database implemented with the blastn algorithm (max E-value 1e^−30^). Singletons, intended as reads present once in the entire sequence dataset (Zhang et al. 2015; Fernández-Mendoza et al. 2017; Moya et al. 2017), were removed. Our workflow (Fig. 2) was organized into three steps in which we analyze progressively more reduced datasets of reads. All the reads representing the mycobiont hosts of each sample at genus level (e.g., *Lecanora*, *Rhizocarpon*) were subtracted from the initial complete dataset. This reduced second dataset was named “no host.” From the “no host” dataset, all reads corresponding to lichen mycobionts (i.e., *Caloplaca*, *Parmelia*), independently from their presence in the lichen community under study, were further filtered out and the obtained third dataset was named “no myco.”

Due to the lack or the limited number of reference sequences in the NCBI database for certain lichen host species, such as *Aspilidea myrinii*, *Psorinia conglomerata*, and *Varicellaria lactea*, the automatic blast search resulted in an incorrect taxonomic assignment of these taxa. They matched with sequences of the genera *Cladonia*, *Lecania*, and *Mycosphaerella*, respectively. Furthermore, the automatic blast search resulted in “no blast hit, unclassified” for a number of OTUs corresponding to the lichen host *Rhizocarpon*; these biases were manually corrected. Our dataset included 13 samples that were symptomatically infected by ten species of lichenicolous fungi (Fig. 1, Table 1, and Table 2). However, ITS reference sequences were available in NCBI database for only four genera, *Arthonia*, *Endococcus*, *Skyttea*, and *Taeniolella*, and corresponded to the following hits: *Arthonia sardoa* (AF138813), *Endococcus fusigera* (FJ645262), *Skyttea gregaria* (KJ559537), *S. radiatilis* (KJ559536, KJ559538), *S. lecanorae* (KJ559539), *S. cismonicae* (KP984783), *Taeniolella stilbospora* (AY843127), *T. phialosperma* (GU966521, KF703925, LC053497), and *T. rudis* (JQ429152). Read identity of the three lichenicolous fungi *Endococcus macrosporus*, *Skyttea tephromelarum*, and *Taeniolella atricerebrina* in the respective symptomatically infected samples (Table 2) could be confirmed according to the reference sequences.

**Table 2:** Number of OTUs and corresponding number of reads identified as the lichen host and the symptomatic lichenicolous fungi (in bold)

Sample	Lichen host	OTUs (reads)	Infecting fungus	A. varians*	*C. epipolytropa*	E. macrosporus*	*L. lecanorae*	*M. atricola*	*M. pygmaea*	*S. eucline*	*S. fissurisedens*	S. tephromelarum*	T. atricerebrina*
A032^#^	*Umbilicaria cylindrica*	76 (11,681)	–										
A138	*Candelariella vitellina*	200 (10,937)	–										OTU52 (3)
A172^#^	*Rhizocarpon geographicum*	46 (12,428)	–										
A194	*Rhizocarpon geographicum*	112 (13,570)	*Endococcus macrosporus*			**OTU6656 (48)**							
A227	*Lecanora swartzii*	527 (12,274)	–										OTU52 (1)
A229	*Psorinia conglomerata*	85 (10,830)	–								OTU43 (2)		
A243^#^	*Lecanora polytropa*	71 (12,952)	–										
A280°	*Tephromela atra*	111 (6277)	*Skyttea tephromelarum*								OTU43 (1)	**OTU3878 (2)**	
A360	*Lecanora intricata*	52 (6658)	–	–									OTU52 (4)
A361^#^	*Tephromela atra*	115 (10,996)	–										
A368	*Lecanora bicincta*	203 (11,800)	–							OTU45 (1)			
A405	*Rhizocarpon geographicum*	33 (9586)	*Muellerella pygmaea-Rg*						**–**	OTU45 (1)			
A418°	*Lecanora polytropa*	52 (11,608)	*Lichenoconium lecanorae*				**OTU15 (36)**				OTU43 (2)		
A420	*Aspilidea myrinii*	234 (7554)	–								OTU43 (238)		
A434°	*Lecanora polytropa*	22 (3926)	*Lichenoconium lecanorae*				**OTU15 (1208)**				OTU43 (1)		OTU52 (1)
A440°	*Tephromela atra*	86 (7905)	*Muellerella atricola*					**OTU4764 (111)**					OTU52 (7)
A476	*Psorinia conglomerata*	65 (9864)	–							OTU45 (1)			
A482	*Lecanora polytropa*	87 (8700)	*Cercidospora epipolytropa*		**OTU8283 (123)**								
A608	*Aspilidea myrinii*	204 (4362)	*Sagediopsis fissurisedens*								**OTU43 (7312)**		
A622°	*Varicellaria lactea*	122 (6772)	*Stigmidium eucline*							**OTU45 (3288)**			OTU52 (3)
A623	*Acarospora fuscata*	77 (15,901)	–										OTU52 (1)
A636	*Lecidea lapicida*	15 (224)	*Muellerella pygmaea s.s.*						**OTU38 (40)**				
A670°	*Lecanora polytropa*	52 (2476)	*Muellerella pygmaea-Lp*						**OTU38 (4633)**				OTU52 (1)
A792	*Lecidea lapicida*	56 (11,463)	–								OTU43 (1)		
A809	*Tephromela atra*	106 (11,373)	*Taeniolella atricerebrina*										**OTU52 (7093), OTU3 (214), OTU1403 (49)**
A832	*Lecanora bicincta*	141 (7048)	*Arthonia varians*	**–**									

°Samples in which reads corresponding to more than one lichenicolous fungus were recovered

^#^Samples without reads corresponding to any lichenicolous fungi

*Lichenicolous fungi for which the identity was confirmed with GenBank BLAST search

Statistics and ecological indices were performed with QIIME (Caporaso et al. 2010). The alpha and beta diversity analyses were conducted on the three datasets (i.e., complete dataset, “no host,” “no myco”) each rarefied to the lowest read count, considering samples with at least 1000 reads. Alpha diversity was calculated using Chao1 (Chao et al. 2009) and Shannon indices (Spellerberg and Fedor 2003). The non-parametric Kruskal-Wallis test was applied to verify the significance of differences in alpha diversity between symptomatically infected and asymptomatic samples with R v.3.2.0 (R Core Team 2015). The distribution of beta diversity was explored using principal coordinate analysis (PCoA) on Bray-Curtis distance matrices; the uncertainty in PCoA plots was estimated using jackknife replicates. Rarefaction was applied by taking a random subset of reads for each sample, corresponding to the 80% of the total read number of those samples with the lowest number of reads in each dataset. The PCoA axes were visualized with EMPeror (Vazquez-Baeza et al. 2013) in two-dimensional plots. Spearman’s correlation on the samples was performed using the software package Statistica v.10 (StatSoft Inc.) to verify the linear relationship between the taxonomic compositions detected by ITS1 (Fernández-Mendoza et al. 2017) and by ITS2 (this study) barcodes.

Shared OTUs were visualized using the software Circos v.0.63-9 (Krzywinski et al. 2009). We compared (i) the amount of shared OTUs among samples, considering the complete, the “no host,” and the “no myco” datasets; (ii) the mycobiomes in the “no myco” dataset among samples of the same lichen host genus or species (*Lecanora* spp., *Rhizocarpon geographicum*, and *Tephromela atra*) which were either symptomatically infected by different lichenicolous fungi or asymptomatic; and (iii) the presence of the main orders of lichen-associated fungi in symptomatically infected and asymptomatic samples.

### DNA extraction, amplification, and sequencing of fungal isolates

To determine whether any fungus isolated in culture from lichen samples within the same community was also detected in the amplicon dataset, we selected ten fungal isolates available from the previous analyses of Muggia et al. (2016). The ten isolates (A572, A899, A923, A930, A931, A951, A985, A993, A1022, A1033) represent those strains of Dothideomycetes and Eurotiomycetes which were most frequently isolated from the studied lichen community. The DNA was extracted with the Plant DNeasy Kit (Qiagen) following the manufacturer’s instructions. The fungal nuclear ribosomal ITS2 region was amplified with the forward primer ITS3 and the reverse primer ITS4 (White et al. 1990). The PCR reaction mix contained 3 μl DNA template (10–20 ng), 5 μl Taq Buffer A (10×, Kapa Biosystems), 0.2 μl Taq DNA Polymerase (5 U/μl, Kapa Biosystems), 1 μl dNTPs (10 mM), 2 μl forward primer ITS3 (10 μM), and 2 μl reverse primer ITS4 (10 μM) in a final volume of 50 μl. The PCR amplifications were performed with the following cycling profile: 95 °C for 3 min and 38 cycles at 95 °C for 30 s, 55 °C for 30 s, 72 °C for 1 min followed by a final extension at 72 °C for 1 min. A negative control was used to verify the absence of non-specific amplification products along the whole amplification and sequencing process. Sanger sequencing of PCR products (one for each culture) was performed at the Applied Genomic Institute (IGA) in Udine (Italy).

## Results

### DNA sequencing and data analysis

A total of 422,740 raw reads with an average length of 342 bp were generated after quality filtering (Table 1); raw data can be accessed at the NCBI short read repository under the accession number SRR5750451. After the extraction of ITS2 and checking for chimera sequences, 297,693 reads were retained to constitute the complete dataset. The sequencing depth was not even among samples, ranging between 7133 and 19,353 reads, with only one sample with less than 1000 reads (Table 1). After excluding reads belonging to the mycobiont host in each sample, 58,528 reads were retained to constitute the “no host” dataset. The subsequent exclusion of reads belonging to any lichen mycobionts from all the samples retained 49,811 reads to form the “no myco” dataset (Fig. 2, Table 1).

Rarefaction curves of the three datasets showed large variation in the total number of OTUs among samples; not all of them leveled off and approached saturation, indicating that detection of additional OTUs may be possible (Supplementary Fig. 1).

### Comparison between symptomatically infected and asymptomatic samples

The complete dataset was rarified to 7133, the “no host” to 1073, and the “no myco” to 1060 reads. This led to the progressive exclusion of one (A636), 13 (A032, A227, A243, A280, A361, A368, A418, A420, A482, A623, A636, A792, and A832), and 14 samples (A032, A227, A243, A280, A361, A368, A418, A420, A476, A482, A623, A636, A792, and A832) from the three datasets, respectively. Sample A434 (*Lecanora polytropa* infected with *Lichenoconium lecanorae*) presented the highest fungal diversity in all three datasets (579 ± 73, 426 ± 148, and 400 ± 158 in the complete, “no host” and “no myco” dataset, respectively) according to the Chao1 index, and the highest diversity only in the complete dataset, according to the Shannon diversity index (5.06 ± 0.11). No significant differences between infected and asymptomatic samples were found in the three datasets (Chao1 *p* values 0.302, 0.685, and 0.540; Shannon *p* values 0.625, 0.306, and 0.882 for the complete, “no host,” and “no myco” datasets respectively; Table 1) when the indexes were compared with the Kruskal-Wallis test.

**Fig. 3:**
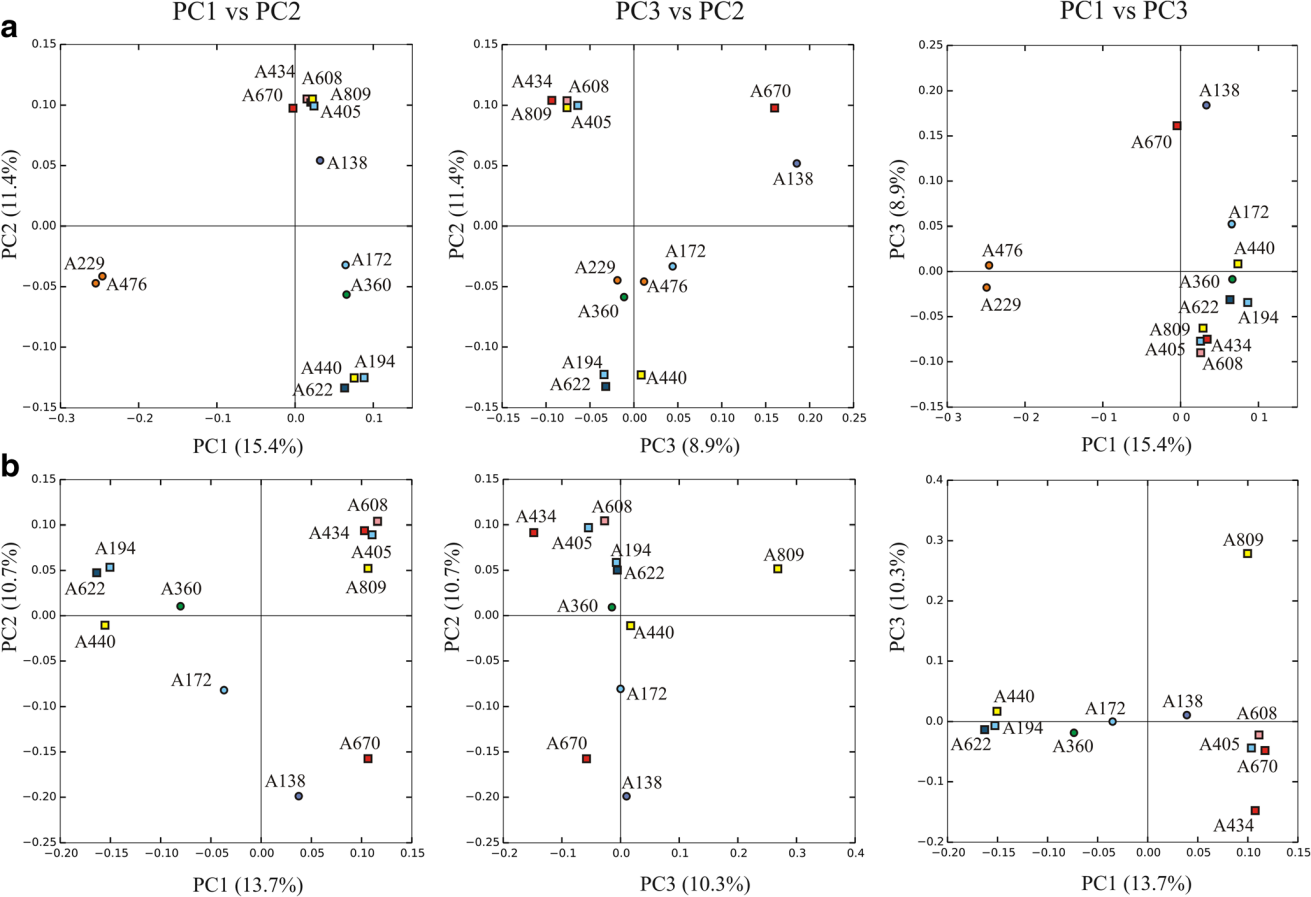
Principal coordinate analysis (PCoA) plots of Bray-Curtis distances based on the rarefied datasets of “no host” (A) and “no myco” (B). Symptomatically infected samples are represented by squares; asymptomatic samples are represented by circles. The percentage of variation explained by each axis is reported in parentheses. The colors indicate different lichen hosts: *Aspilidea myrinii* (pink), *Candelariella vitellina* (violet), *Lecanora intricata* (green), *L. polytropa* (red), *Psorinia conglomerata* (orange), *Rhizocarpon geographicum* (light blue), *Tephromela atra* (yellow), and *Varicellaria lactea* (blue). Samples ID are as in Table 1. PC, principal coordinate

The beta diversity analysis showed that, in the complete dataset, samples were grouped mostly according to the lichen host species. Here, samples of *Psorinia conglomerata* and *Tephromela atra* distinctly separate from the other samples (Supplementary Fig. 2). In the PCoA analyses of the “no host” (Fig. 3(A)) and “no myco” (Fig. 3(B)) datasets, the maximum percentage of variation explained by PC1 axis was 15.4 and 13.7%, respectively (Fig. 3 and Supplementary Fig. 3). The two-dimensional plots in both datasets do not separate the samples according to the lichen host, the lichen-associated fungi, or the symptomatic fungal infection.

### Detection of fungal diversity: lichen mycobionts and lichen-associated fungi

**Fig. 4:**
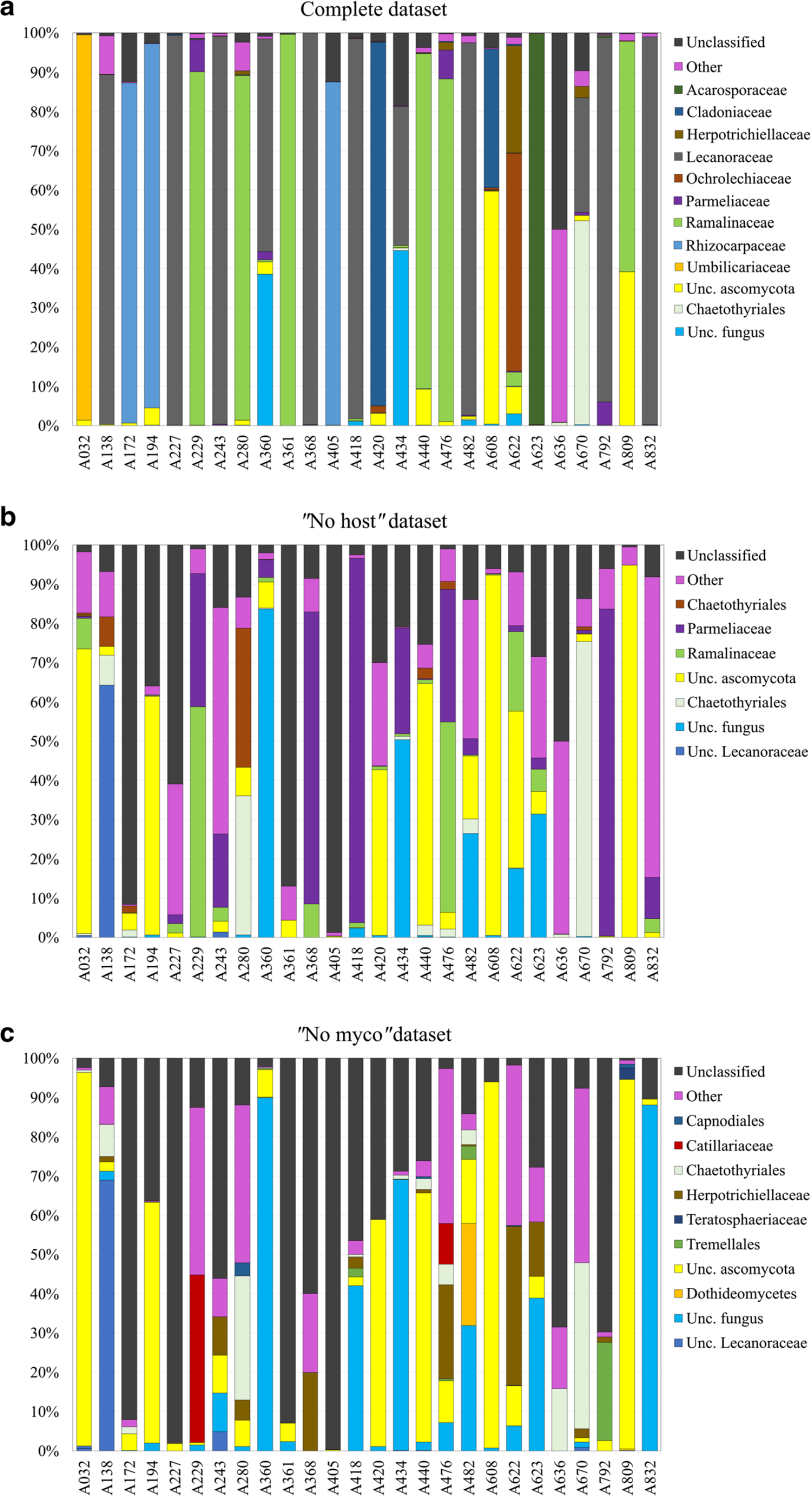
Summary of the taxonomic assignment up to family level of the complete (**a**), the “no host” (**b**), and the “no myco” (**c**) datasets. Taxa accounting for < 1% (in **a** and **b**) and < 0.1% (in **c**) of reads are grouped as “Other.” “Unc.” stays for “uncultured.” Bars reflect the proportion of reads from the ITS2 dataset for each sample. Samples ID are as in Table 1

Almost all the reads assigned at kingdom level were ascomycetes (99.9%, Fig. 4); basidiomycetes (mostly Tremellomycetes) were detected in a very low proportion and in 12 samples only. In 12 samples, over 90% of the reads corresponded to the lichen mycobiont (Fig. 4a, Table 1, and Table 2). The three samples that were symptomatically infected by lichenicolous fungi (A434, A608, and A670) also had the lowest proportion of mycobiont reads (< 35%). In each sample, multiple OTUs were found to correspond to the same mycobiont host (as similarly recovered by Fernández-Mendoza et al. 2017), whereas for the lichenicolous fungi this was the case only for *Taeniolella atricerebrina*, for which three OTUs were recovered (Table 2).

*Taeniolella atricerebrina* was detected asymptomatically in samples of the same lichen host (*Tephromela atra* A440) symptomatically infected by the lichenicolous fungus *Muellerella atricola*, and in other four lichen hosts (*Acarospora*, *Candelariella*, *Lecanora*, *Varicellaria*; Table 2). *Taeniolella atricerebrina* was identified by three OTUs, the most abundant represented by 7093 reads (OTU52), the second and the third most abundant ones by 214 and 49 reads (OTU3 and OTU1403), respectively. All three OTUs were present in the symptomatically infected sample *Tephromela atra* A809, while only the most abundant OTU52 was recovered in the other samples, though with a number of reads ranging from 1 to 7 (Table 2).

Based on the abundance and taxonomic assignment, we predicted the identity of the reads corresponding to the symptomatically infecting lichenicolous fungi *Cercidospora epipolytropa* (A482), *Stigmidium eucline* (A622), *Lichenoconium lecanorae* (A418, A434), *Sagediopsis fissurisedens* (A608), and *Muellerella atricola* (A440). For each of these fungi, a blast search recovered a single OTU matching with “uncultured Ascomycota” or “unclassified.” The OTUs corresponding to *Sagediopsis fissurisedens* (OTU43) and *Stigmidium eucline* (OTU45) were also found in lichen samples other than their known hosts (Table 2). The lichenicolous fungus *Muellerella pygmaea* was symptomatically present in three lichen samples (A405, A636, A670); however, OTU38, which we tentatively assigned to *M. pygmaea* because it matched with Chaetothyriales in a blast search, was found only in two of them (in A636 with 40 reads, 9%; in A670 with 4633 reads, 52%). This result suggests that the identification of *Muellerella* could be correct, as previous studies reported the fungus in this order (Muggia et al. 2015; Triebel and Kainz 2004). In the single case of the sample *Lecanora bicincta* A832 infected by *Arthonia varians*, we could not detect any OTU assignable to the lichenicolous fungus. Finally, we did not recover any OTU assignable to lichenicolous fungi in four specimens (A032, A172, A243, A361), whereas we recovered OTUs of different lichenicolous fungi co-occurring in seven specimens, of which six were symptomatically infected (A280, A418, A434, A440, A622, A670) and one was without visible infection (A360; Table 2).

In the “no host” dataset, 23% of the reads belonged to the orders Chaetothyriales (Eurotiomycetes, Ascomycota) and Lecanorales (Lecanoromycetes, Ascomycota) (Fig. 4b). The most represented families were Herpotrichiellaceae (Chaetothyriales), Parmeliaceae, and Ramalinaceae (Lecanorales); 30% of the reads could be assigned up to the kingdom level (Fig. 4b). Reads blasting as “uncultured fungi” and “unclassified” represented 13 and 15% of the dataset, respectively.

In the “no myco” dataset (Fig. 4c), up to 37% of the reads could be assigned to the order level within Ascomycota and they belonged again to Chaetothyriales (Eurotiomycetes), Capnodiales (Dothideomycetes), and Lecanorales (Lecanoromycetes). The most represented families were Herpotrichiellaceae (Chaetothyriales) and Catillariaceae (Lecanorales). About 0.12% belonged to Tremellales (Basidiomycota), 22% to “uncultured fungi,” and 17% remained unclassified (Fig. 4c).

**Fig. 5:**
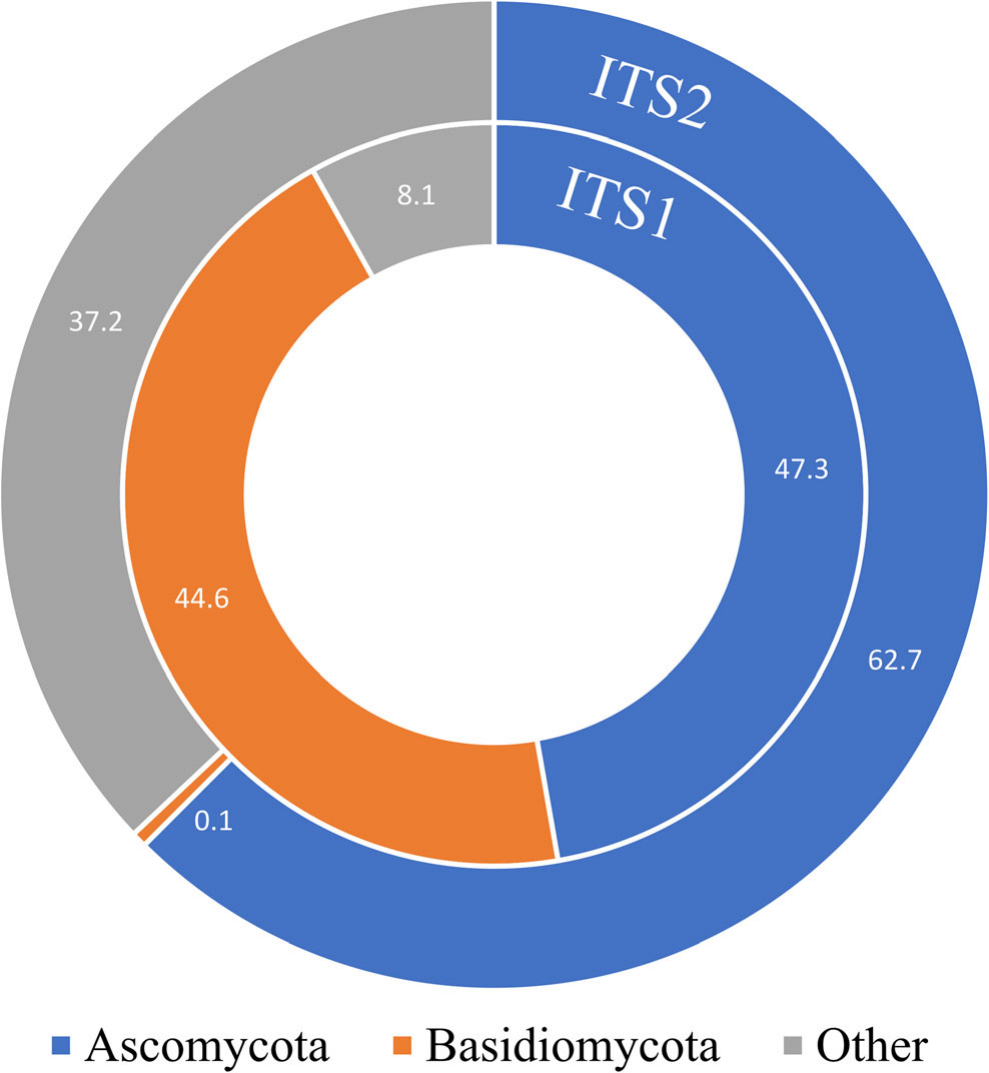
Comparison between the taxonomic composition of ITS1 (Fernández-Mendoza et al. 2017) and ITS2 datasets based on the most recovered fungal orders in Ascomycota (Capnodiales and Chaetothyriales) and Basidiomycota (Tremellales). Bars reflect the proportion of reads (expressed in percentage) assigned to the respective taxa in the two datasets. “Other” comprehends other fungal divisions, uncultured and unidentified fungi

The relative abundances of Ascomycota and Basidiomycota among the lichen-associated fungi were compared (Fig. 5) between the ITS1 (Fernández-Mendoza et al. 2017) and ITS2 datasets (this study). Spearman’s correlation was calculated for the most represented orders Capnodiales, Chaetothyriales, and Tremellales. The relative abundances were 0.24, 0.07, and − 0.036 respectively and indicated no significant (*P* < 0.05) linear relationship between ITS1 and ITS2 datasets. The relative abundance of these orders differs between the two barcodes, being 25.6 and 0.3% for Capnodiales, 10.1 and 9.6% for Chaetothyriales, and 44.5 and 0.2% for Tremellales in the ITS1 and ITS2 datasets, respectively.

### Shared OTUs among samples

**Fig. 6:**
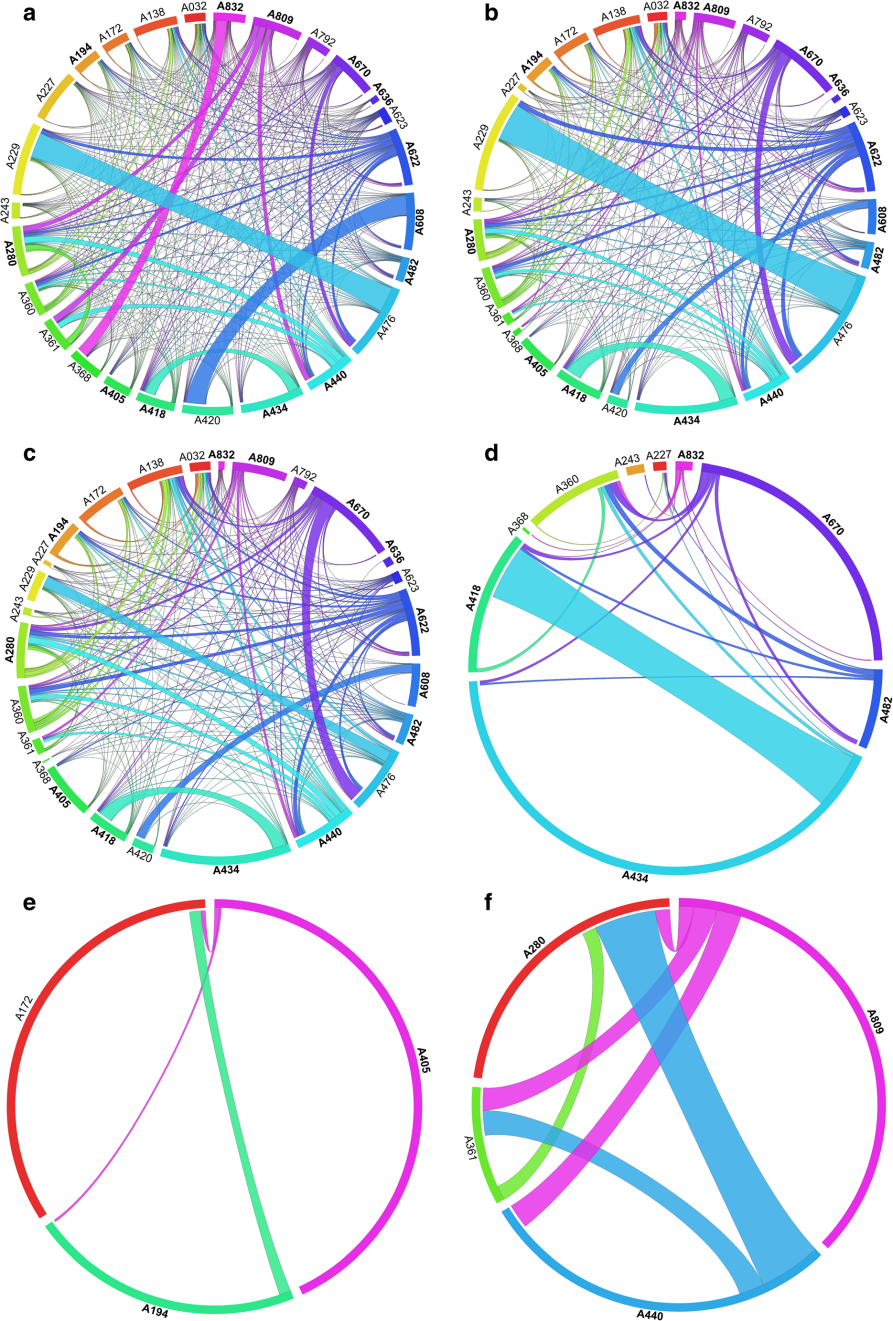
Circos plots showing shared OTUs among lichen mycobiomes. Symptomatically infected samples (as in Table 1) are in bold. The length of the sample ribbons is directly proportional to the number of OTUs identified in each sample. The width of each connector between two samples is directly proportional to the number of shared OTUs. Shared OTUs among all samples in the complete dataset (**a**), “no host” dataset (**b**), and “no myco” dataset (**c**) are presented. Shared OTUs calculated on the “no myco” dataset among samples of the same mycobiont genus or same species are shown for the lichens *Lecanora* spp. (**d**), *Rhizocarpon geographicum* (**e**), and *Tephromela atra* (**f**)

**Fig. 7:**
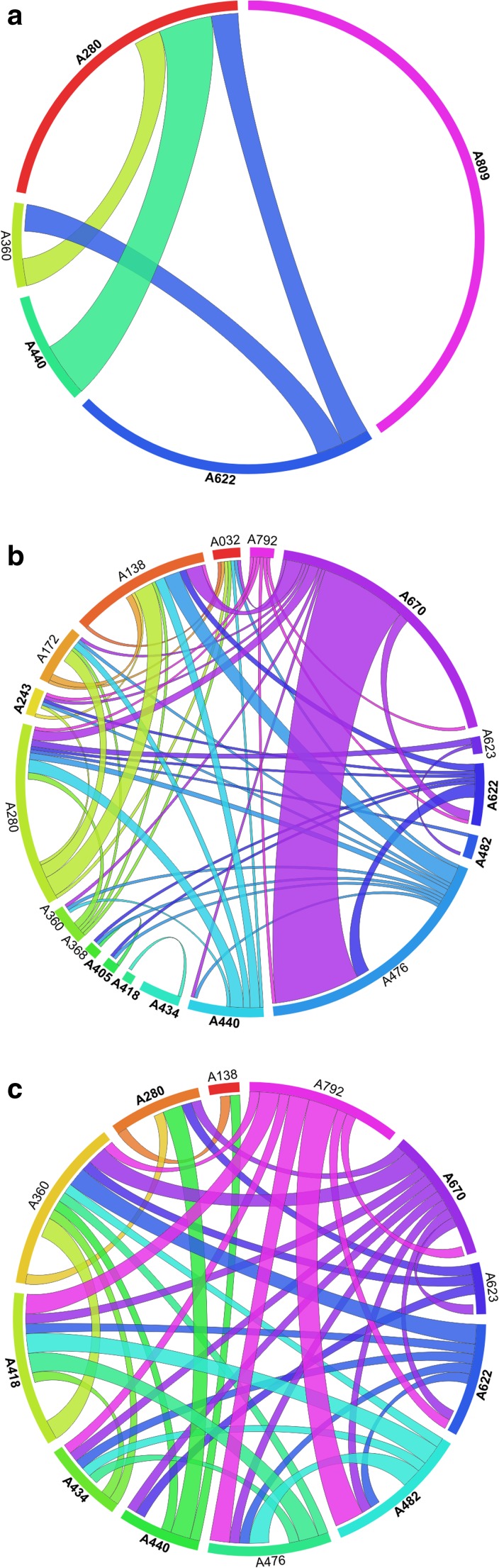
Circos plots showing shared OTUs among lichen mycobiomes. Symptomatically infected samples (as in Table 1) are in bold. The length of the sample ribbons is directly proportional to the number of OTUs identified in each sample. The width of each connector between two samples is directly proportional to the number of shared OTUs: **a** Capnodiales, **b** Chaetothyriales, and **c** Tremellales

Though each sample is characterized overall by a high proportion of sample-specific OTUs, lichen mycobiomes are quite interconnected due to many shared OTUs (Fig. 6; Supplementary Tables 1–9). The main orders of lichen-associated fungi in which shared OTUs are recovered are Capnodiales, Chaetothyriales, and Tremellales (Fig. 7; Supplementary Tables 7–9).

In the complete dataset (Fig. 6a; Supplementary Table 1) and in the “no host” dataset (Fig. 6b; Supplementary Table 2), the two samples A229 and A476 of *Psorinia conglomerata* share a maximum of 307 and 250 OTUs, respectively. The 250 shared OTUs in *P. conglomerata* belong mainly to mycobiont genera of Ramalinaceae and Parmeliaceae and are responsible for the strong similarity of the two samples (as in Fig. 3(A)). No OTUs were shared by 26 pairs of samples in the complete dataset nor by 102 pairs of samples in the “no host” dataset.

In the “no myco” dataset (Fig. 6c; Supplementary Table 3), a maximum of 60 shared OTUs between the two samples A418 and A434 of *Lecanora polytropa* was recorded. This redundancy was seen also in the analysis comparing the OTUs’ diversity among *Lecanora* spp. samples only (Fig. 6d). The 60 OTUs belong mostly to unclassified and uncultured fungi and include reads that we predict to be the lichenicolous fungus *Lichenoconium lecanorae* (Table 2; Supplementary Table 4). This is supported by the symptomatic presence of *Lichenoconium lecanorae* on both A418 and A434 *L. polytropa* samples. In the “no myco” dataset, no OTUs were shared by 132 pairs of samples.

In the three samples of *Rhizocarpon geographicum* (Fig. 6e), the asymptomatic sample A172 shared two OTUs with sample A405 symptomatically infected by *M. pygmaea*, and five OTUs with sample A194 symptomatically infected by *E. macrosporus*. The two symptomatically infected samples A194 and A405 shared only one OTU (Supplementary Table 5).

*Tephromela atra* A361 without symptomatic infection shared OTUs with all the other symptomatically infected thalli of *T. atra* (Fig. 6f): seven OTUs with sample A280 infected by *S. tephromelarum*, 12 OTUs with sample A440 infected by *M. atricola*, and 11 OTUs with A809 infected by *T. atricebrina*. The three symptomatically infected *T. atra* were connected with a minimum of seven and a maximum of 30 shared OTUs. Samples A280 and A809 shared the same OTU of *T. atricebrina*, which is therefore detected as asymptomatic in A280 (Table 2). Samples A280 and A440 share 30 OTUs mostly belonging to “uncultured Ascomycota” (Supplementary Table 6).

Capnodiales (Fig. 7a) were present in five samples, of which five were infected symptomatically and two asymptomatically. No more than two shared OTUs were detected between the two symptomatically infected samples A280 (*T. atra* infected by *S. tephromelarum*) and A440 (*T. atra* infected by *M. atricola*).

Chaetothyriales (Fig. 7b) were present in 17 samples (eight symptomatically infected and nine asymptomatic) and a maximum of 26 OTUs were recorded between samples A476 (*P. conglomerata*) and A670 (*L. polytropa* infected by *M. pygmaea*).

Tremellales (Fig. 7c) were present in 12 samples (seven symptomatically infected and five asymptomatic) and a maximum of three OTUS were shared between the symptomatically infected sample A482 (*L. polytropa* infected by *C. epipolytropa*) and the asymptomatic sample A792 (*Lecidea lapicida*).

### Amplicon sequencing vs. fungal isolates results

The ten selected fungal strains were all amplified for the ITS fragment; however, ITS2 sequences could be successfully obtained only for four of them (NCBI accessions MF276907-MF276910) and were queried against the complete dataset. ITS2 sequences of the strains A930 and A1022 successfully matched (≥ 97%) with a total of five OTUs (Supplementary Table 10). The cultured strain A923 is a Dothideomycete (Lichenostigmatales; Muggia et al. 2016) isolated from a thallus of *T. atra* symptomatically infected by *M. atricola*; it matched with two OTUs of “uncultured Ascomycota” in 14 samples. These included both multiple lichen hosts and the sample A440, which represents the same combination of mycobiont-lichenicolous fungus (*T. atra* infected by *M. atricola*) of the thallus used for the isolation of this fungus. The strain A1022 is a Eurotiomycete (Chaetothyriomycetidae; Muggia et al. 2016) and was isolated from a thallus of *R. geographicum* symptomatically infected by *E. macrosporus*. The three matching OTUs were assigned to the group of “fungal endophyte” and were present in two samples (Supplementary Table 10). In this case, however, there is no correspondence with the lichen used for the isolation, as the detected OTUs came from two *Lecanora* spp. specimens (A360 and A832).

## Discussion

### Lichen mycobiome diversity

Though the comparison between ITS1 and ITS2 barcoding markers is not novel for fungal communities, it has not been tested for lichens yet, and it gives here pioneering insights for methodological approaches in studying lichen mycobiomes.

Because the two datasets of the ITS1 and the ITS2 were gained independently, using two different sequencing approaches and clustering algorithms, we have refrained from comparing them more closely. Alternatively, we opted to compare the taxonomic diversity as far as possible and to comment on the differential detection of taxa. Our approach, which considers the lichen thallus as distinctive and still largely unexplored niche for unknown fungal assemblages, further strengthens the perception that diversity estimates based on metabarcoding are limited by the barcode locus selected (Tedersoo et al. 2015; Tedersoo and Lindahl 2016).

Our workflow (Fig. 2) was organized into three steps that analyzed a progressively more reduced dataset of reads. With this method, we succeeded in reliably assessing the fungal diversity of each sample at different taxonomic levels, and in predicting which reads potentially correspond to the symptomatically infecting lichenicolous fungi. This enabled comparison of symptomatically infected and asymptomatic samples using alpha and beta diversity indexes.

Alpha diversity in symptomatically infected samples is not higher than that in lichens devoid of fungal infections. Beta diversity was characterized by the low percentage of variation explained by the three major axes (around 35% in total for both “no host” and “no myco” datasets). Moreover, due to the rarefaction of the datasets, the results are impaired by the number of retained samples. Indeed, symptomatically infected and asymptomatic samples are unequally represented, being the asymptomatic samples only three out of 11 samples in the “no myco” dataset. Overall, the beta diversity analyses showed no tendency among samples to group according to presence/absence of symptomatic infection nor according to lichen host species. This observation is in congruence with the results presented by Fernández-Mendoza et al. (2017).

The presence of different haplotypes derived from different fungal individuals could explain why multiple OTUs for the same mycobiont species were recovered. On rocks, lichen thalli develop side by side, and hyphae from one mycelium could penetrate into neighboring thalli. The multicopy nature of the ITS region (Schoch et al. 2012) may also result in an overestimation of diversity if divergent paralogs or non-orthologous gene copies are sequenced (Simon and Weiß 2008; Lindner and Banik 2011). However, this intragenomic variation does not compromise the taxonomic identification value of the ITS region (Hollingsworth 2011). Another, more parsimonious explanation that cannot be ruled out in any sequencing approach is that errors may be introduced by sequencing.

The main orders of lichen-associated fungi detected by the ITS2 barcode were Capnodiales, Chaetothyriales, and Tremellales (Basidiomycetes), which closely agrees with the results obtained previously by analyzing the ITS1 fragment. The order Capnodiales includes endophytes, pathogens, and, like Tremellales, parasites of fungi (Crous et al. 2009; Lindgren et al. 2015). Chaetothyriales are saprobic, rock-inhabiting, lichenicolous, and epiphytic fungi (Réblová et al. 2013; Lawrey and Diederich 2016). In our dataset, these orders are distributed differently among the samples and do not show any correlation with the lichen host species or the presence of symptomatic infections. The same pattern is observed for the relevant fraction of unidentified taxa (i.e., uncultured Ascomycota, uncultured fungus, unidentified), which could belong to parasymbiontic or commensal fungi occurring incidentally on lichen thalli, as hypothesized by Fernández-Mendoza et al. (2017).

### ITS barcodes capture unequal taxon diversity in lichen mycobiomes

Lichen mycobiomes are still uncharted terrains for investigating patterns of fungal specificity and ecological adaptations and have recently become the subjects of metabarcoding analyses (Bates et al. 2012; U'Ren et al. 2014; Zhang et al. 2015; Mark et al. 2016). In our sequencing of the ITS2 locus, the proportion of reads belonging to the lichen hosts is higher (min 27.7%, max 99.8%) than those obtained previously from ITS1 (min 3.5%, max 97.7%; Fernández-Mendoza et al. 2017). Although we could not assign any reads to two lichenicolous fungal species, *M. pygmaea* and *A. varians*, using either ITS1 (Fernández-Mendoza et al. 2017) or ITS2, with ITS2 we were able to detect reads assignable to other lichenicolous taxa in asymptomatic thalli. Chaetothyriales and Capnodiales are the most highly represented orders detected in lichen mycobiomes using both ITS1 and ITS2 barcodes.

Basidiomycetes are known to be common partners in lichen symbioses (Spribille et al. 2016; Oberwinkler 2017). The previous study, performed with 454 pyrosequencing and based on the ITS1 barcode, demonstrated a high proportion of Tremellomycetes in the samples, with basidiomycetes present in 23 and representing the main component of 11 samples (Fernández-Mendoza et al. 2017). On the other hand, basidiomycetes were the least detected in our dataset: they were represented by less than 1% of all the reads and were found in only 12 samples. Other studies reported a variable fraction of basidiomycetes in lichen mycobiomes: about 15% of the complete dataset in arctic lichens (Zhang et al. 2015; analyzing the whole ITS region), less than 1% of rock-inhabiting foliose lichens (Bates et al. 2012; 18S rRNA) and less than 3% among endolichenic fungi in a comprehensive study (U’Ren et al. 2012; analyzing the complete ITS region). It is important to note that these studies considered lichens with growth forms (foliose and fruticose) different from those in the community we studied (epilithic and crustose thalli). Implicitly, lichen growth forms likely influence the presence of certain fungal taxa within the thalli.

### ITS1 vs. ITS2 as barcode for lichenicolous fungi

Given that the selected samples harbored symptomatic lichenicolous fungi and a high proportion of other asymptomatic fungi (Fleischhacker et al. 2015; Fernández-Mendoza et al. 2017; Muggia et al. 2016), particular attention was paid in predicting which sequences, based on their read abundance and taxonomic assignment, could represent the symptomatic lichenicolous fungi. Fernández-Mendoza et al. (2017) succeeded in identifying three taxa also found in our analyses.

We identified sequences of potentially five additional lichenicolous fungi (Table 2). We also could detect the same OTUs of three lichenicolous fungal species (*T. atricebrina*, *S. eucline*, and *S. fissurisedens*) in other samples which did not correspond with the known lichen host and occurred asymptomatically. The corresponding reads were found in the samples devoid of symptoms in a much smaller fraction (< 10 reads) than in the symptomatically infected thalli (Table 2); the exception is the high number of reads of *S. fissurisedens* on the asymptomatic host *A. myrinii*. Furthermore, it seems that many lichenicolous fungi can be present in a thallus where only one of them is symptomatically detectable. In this case, the lichenicolous fungus, recognized within the first group of lichen-associated taxa (sensu Fernández-Mendoza et al. 2017) in the symptomatic sample, could be part of the third fungal fraction (sensu Fernández-Mendoza et al. 2017) when its corresponding reads are recovered in the mycobiome of any asymptomatic samples.

Interestingly, the number of reads for each OTU recovered for lichenicolous fungi using ITS2 as barcode is much higher than those recovered using ITS1. As reported by Fernández-Mendoza et al. (2017), also in our analyses, the presence of symptomatic lichenicolous fungi does not affect the composition of the individual lichen mycobiomes in general, but it still remains unexplained if the presence of a lichenicolous fungus may inhibit the symptomatic development of a second one.

The differences in taxonomic composition that emerge when data for either ITS region are analyzed separately suggest that both ITS1 and ITS2 barcodes should be considered together for a more reliable estimation of lichen mycobiome diversity. Monard et al. (2013) reached a similar conclusion for other fungal communities. The application of sequencing platforms that allow analysis of larger fragments, such as PacBio (Pacific Bioscience) or MinION (Oxford Nanopore Technologies), is likely to make the metabarcode sequencing of the whole ITS region feasible in the near future. Also, as it is known that the whole ITS sequence (including ITS1 and ITS2) still does not allow a clear resolution of species in the most common genera of microfungi (e.g., *Aspergillus*, *Colletotrichum*, *Fusarium*; Raja et al. 2017), it may be possible that the sequencing of specific housekeeping genes depending on the genus or even species group within the genus could help in the future to improved species resolution in metabarcoding studies.

### HTS platforms for the analyses of lichen mycobiomes

In the most common environmental samples, such as those from soil or water, the DNA detected and amplified usually contributes evenly to the overall taxonomic composition, regardless of whether animal, plant, fungal, or bacterial barcodes are used (Taberlet et al. 2012; Bálint et al. 2014; Sunagawa et al. 2015; Bell et al. 2016; Vences et al. 2016). Lichen thalli, however, consist mainly of one fungus; when fungal barcodes are analyzed, a high fraction of the reads belong to the lichen mycobiont (Bates et al. 2012; Zhang et al. 2015; Fernández-Mendoza et al. 2017), affecting the sampling depth of the other fungi. This shallow and uneven sampling depth of lichen-associated fungi causes a substantial loss of information, and biases the interpretation of species diversity patterns. This is clearly exemplified by the alpha and beta diversity analyses in our study. About half of the samples in the “no host” and “no myco” datasets had to be excluded due to the low number of reads (< 1000), and the remaining samples did not always approach saturation. This condition is independent from the HTS platform used and could be partially prevented by increasing the complete sampling depth of the analysis, for example, with use of larger PGM chips such as 318™. However, the fraction of lichen mycobiont reads is never predictable. One potential solution would be the use of species-specific blocking primers, which prevent the amplification of non-target DNA. This strategy would substantially increase the cost of the analyses, especially when multiple lichen hosts are excluded from the amplifications. Using multiple blocking primers might further bias the library preparation, as specific blocking oligonucleotides can block closely related non-target sequences at the same time (Leray et al. 2013a; Piñol et al. 2015). This approach has already been used in DNA metabarcoding dietary studies (Deagle et al. 2010; Leray et al. 2013b), where samples are often enriched with the DNA of the host organism (Piñol et al. 2015). If the sequencing depth of the lichen-associated fungi could be selectively increased in metabarcoding studies, it will allow us to significantly deepen the taxonomic and functional analysis of lichen mycobiomes.

## Electronic supplementary material

ESM 1 (PDF 1078 kb)
