# Supplementary material for: ITS2 metabarcoding analysis complements lichen mycobiome diversity data
Source: Mycol Prog. 2018 Jun 22;17(9):1049–66. doi: 10.1007/s11557-018-1415-4 (PMC6428334; doi:10.1007/s11557-018-1415-4)
Supplement: Supplementary file 1 — (PDF 1078 kb) [file 11557_2018_1415_MOESM1_ESM.pdf]

Supplementary material

ITS2 metabarcoding analysis complements lichen mycobiome diversity data

Elisa Banchi[1], David Stankovic[1,2], Fernando Fernández-Mendoza[3], Fabrizia Gionechetti[1], Alberto Pallavicini[1], Lucia Muggia[1]*

Supplementary Fig. 1 Rarefaction curves of the complete (A), “no host” (B) and “no myco” (C) datasets.

A

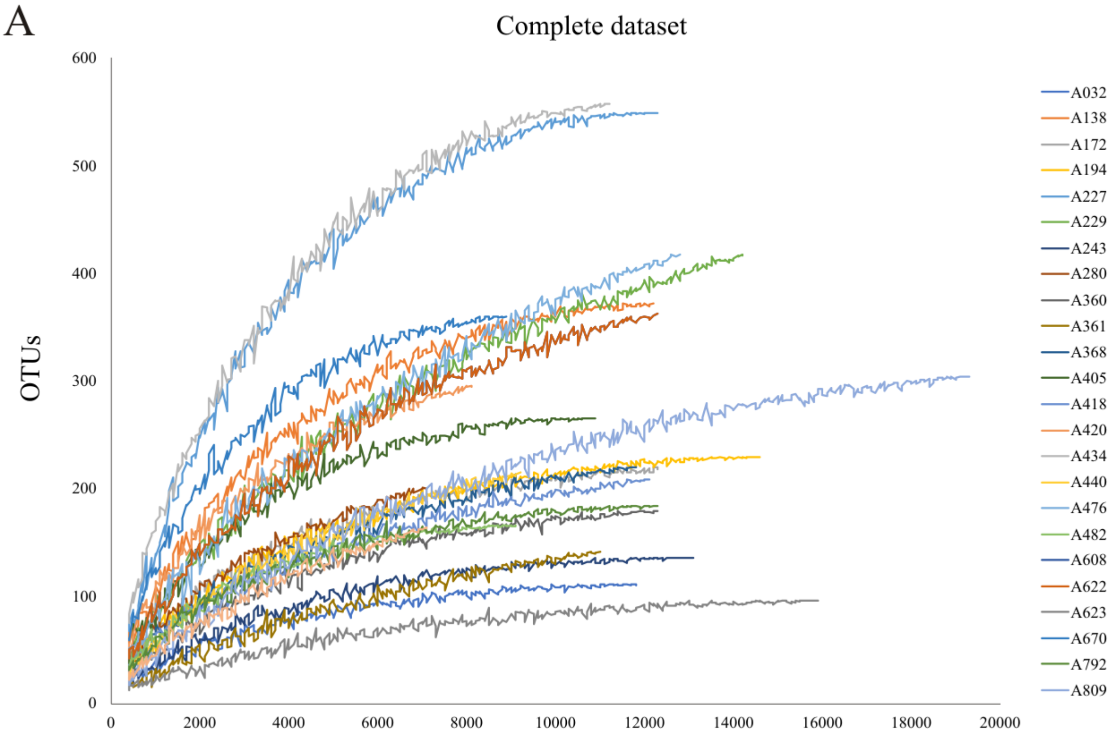

B

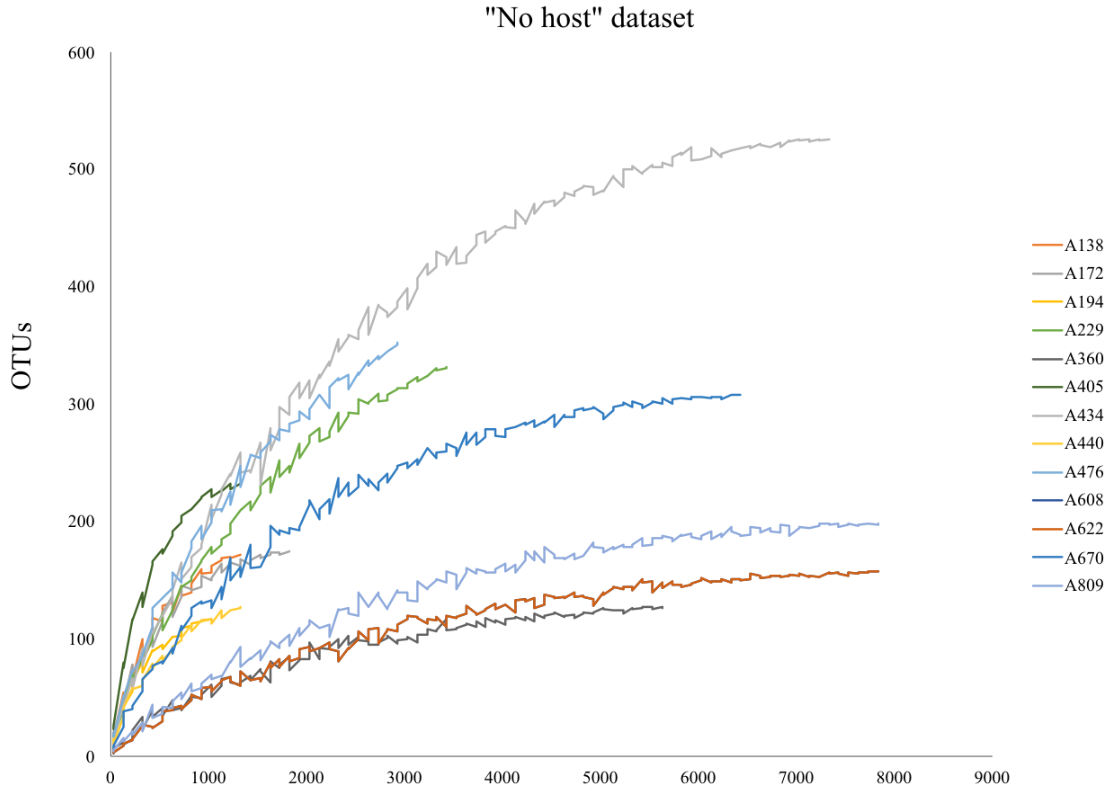

C

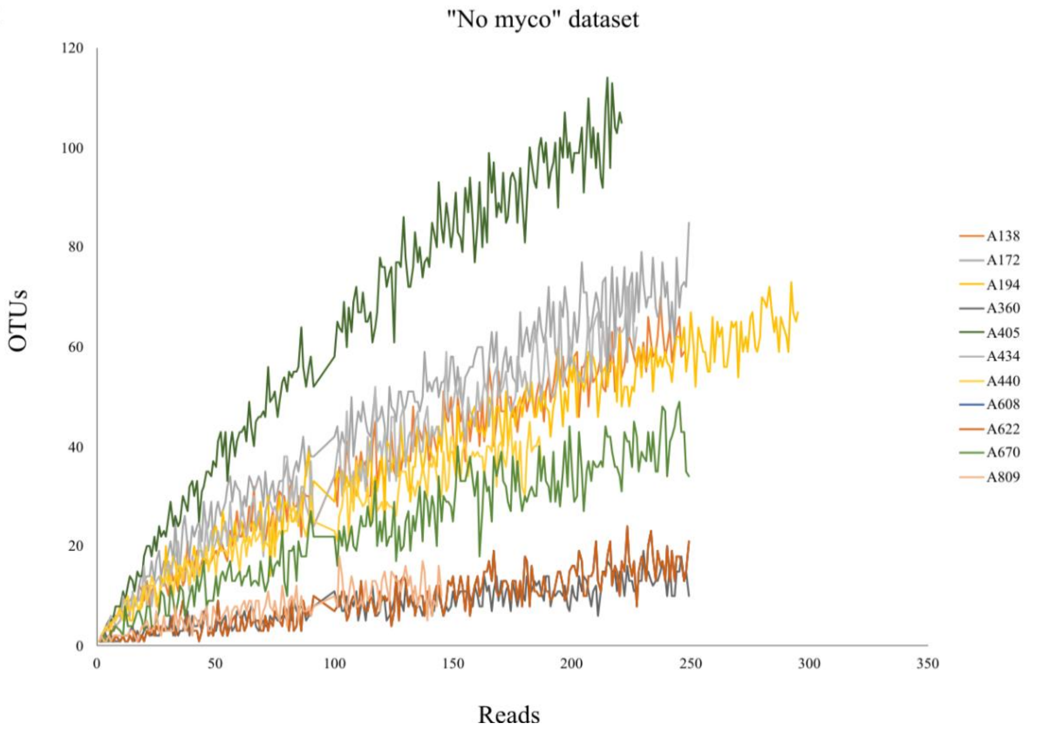
"No myco" dataset
OTUs
Reads
0
20
40
60
80
100
120
0
50
100
150
200
250
300
350
A138
A172
A194
A360
A405
A434
A440
A608
A622
A670
A809

Supplementary Fig. 2 Principal Coordinate Analysis (PCoA) plots of Bray-Curtis distances calculated among the lichen mycobiomes considering the complete dataset.

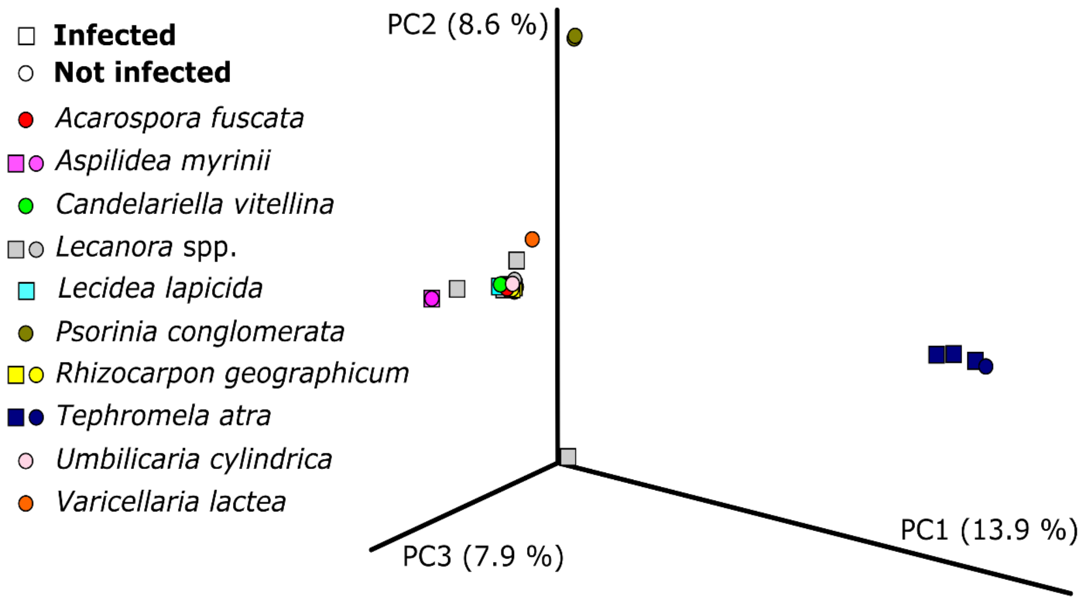

Supplementary Fig. 3 Jackknifed Principal Coordinate Analysis (PCoA) plots of Bray-Curtis distances based on the “no host” (A) and “no myco” (B) datasets. The statistical confidence of the results is presented by ellipsoids around the samples.

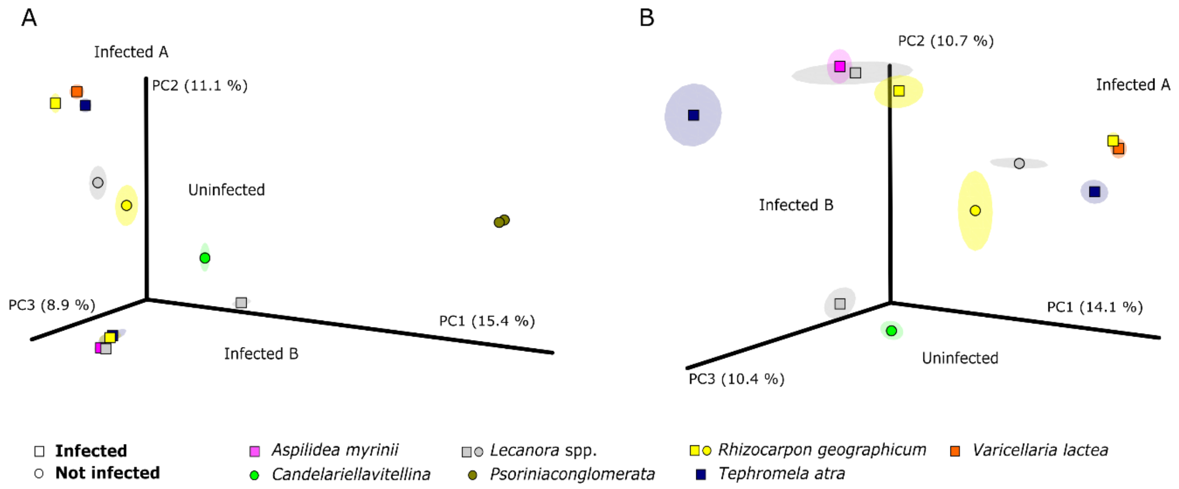

Supplementary Table 1 Matrix of the shared OTUs among samples in the complete dataset used for Fig. 6A.

	A229	A138	A792	A194	A440	A361	A280	A809	A032	A360	A622	A172	A227	A832	A405	A670	A418	A636	A623	A482	A476	A434	A243	A608	A368	A420
**A229**	417	0	5	3	7	5	8	3	3	10	41	4	4	3	3	3	6	2	3	3	307	4	2	5	1	4
**A138**	0	372	3	3	13	3	20	5	7	12	14	5	1	2	1	17	2	0	4	7	14	2	7	0	2	1
**A792**	5	3	184	1	5	1	9	3	3	4	6	1	1	2	2	7	11	1	8	6	4	6	3	7	1	5
**A194**	3	3	1	229	8	1	6	2	6	10	13	7	1	1	2	3	2	0	6	6	1	0	0	1	0	0
**A440**	7	13	5	8	215	69	73	60	14	27	29	8	2	5	3	8	10	1	5	8	7	4	3	4	0	2
**A361**	5	3	1	1	69	141	66	76	3	9	8	1	1	1	1	1	1	1	1	3	3	1	0	1	0	1
**A280**	8	20	9	6	73	66	201	58	12	19	23	11	4	6	3	17	9	1	6	7	8	7	6	4	3	4
**A809**	3	5	3	2	60	76	58	304	8	10	12	2	5	3	2	3	4	1	5	5	2	3	1	1	3	3
**A032**	3	7	3	6	14	3	12	8	111	12	16	9	2	5	4	6	3	1	3	4	3	1	2	1	5	2
**A360**	10	12	4	10	27	9	19	10	12	179	30	10	4	7	4	9	9	1	6	9	10	5	2	1	3	2
**A622**	41	14	6	13	29	8	23	12	16	30	326	9	4	7	9	11	7	3	5	15	42	4	4	17	4	18
**A172**	4	5	1	7	8	1	11	2	9	10	9	221	3	3	5	4	4	1	4	2	2	0	1	0	0	0
**A227**	4	1	1	1	2	1	4	5	2	4	4	3	549	3	4	3	7	1	4	2	0	2	2	1	2	1
**A832**	3	2	2	1	5	1	6	3	5	7	7	3	3	164	2	4	7	2	3	3	1	2	1	5	131	4
**A405**	3	1	2	2	3	1	3	2	4	4	9	5	4	2	265	4	4	1	3	3	1	0	1	0	1	1
**A670**	3	17	7	3	8	1	17	3	6	9	11	4	3	4	4	360	10	2	5	7	55	6	2	2	3	2
**A418**	6	2	11	2	10	1	9	4	3	9	7	4	7	7	4	10	208	2	5	5	3	120	2	9	1	7
**A636**	2	0	1	0	1	1	1	1	1	1	3	1	1	2	1	2	2	52	1	0	0	0	0	1	0	0
**A623**	3	4	8	6	5	1	6	5	3	6	5	4	4	3	3	5	5	1	96	5	1	6	1	2	1	3
**A482**	3	7	6	6	8	3	7	5	4	9	15	2	2	3	3	7	5	0	5	166	8	3	2	4	2	5
**A476**	307	14	4	1	7	3	8	2	3	10	42	2	0	1	1	55	3	0	1	8	417	1	2	0	1	0
**A434**	4	2	6	0	4	1	7	3	1	5	4	0	2	2	0	6	120	0	6	3	1	557	1	4	1	6
**A243**	2	7	3	0	3	0	6	1	2	2	4	1	2	1	1	2	2	0	1	2	2	1	135	2	3	2
**A608**	5	0	7	1	4	1	4	1	1	1	17	0	1	5	0	2	9	1	2	4	0	4	2	362	3	231
**A368**	1	2	1	0	0	0	3	3	5	3	4	0	2	131	1	3	1	0	1	2	1	1	3	3	220	2
**A420**	4	1	5	0	2	1	4	3	2	2	18	0	1	4	1	2	7	0	3	5	0	6	2	231	2	295

Supplementary Table 2 Matrix of the shared OTUs among samples in the "no host" dataset used for Fig. 6B.

	A032	A138	A172	A194	A227	A229	A243	A280	A360	A361	A368	A405	A418	A420	A434	A440	A476	A482	A608	A622	A623	A636	A670	A792	A809	A832
**A032**	35	7	8	6	1	2	0	9	9	1	0	1	2	1	0	10	3	4	0	11	1	0	5	2	4	3
**A138**	7	172	5	3	1	0	4	19	10	2	0	1	2	1	2	13	13	7	0	13	3	0	16	3	4	2
**A172**	8	5	175	5	1	1	0	8	6	0	0	2	1	0	0	6	1	2	0	7	1	0	3	0	0	3
**A194**	6	3	5	117	0	1	0	5	8	0	0	1	1	0	0	6	0	6	1	11	4	0	3	1	0	1
**A227**	1	1	1	0	22	2	0	0	1	0	0	0	0	0	1	1	0	1	0	1	1	0	1	0	1	2
**A229**	2	0	1	1	2	332	0	2	5	2	0	1	2	2	2	3	250	1	2	33	0	0	1	2	1	1
**A243**	0	4	0	0	0	0	64	3	1	0	2	0	1	0	0	3	2	0	0	3	1	0	1	2	0	0
**A280**	9	19	8	5	0	2	3	90	14	7	0	1	5	2	3	31	8	5	1	18	4	0	14	5	8	3
**A360**	9	10	6	8	1	5	1	14	127	2	1	1	5	0	4	21	6	7	0	24	3	0	8	2	3	4
**A361**	1	2	0	0	0	2	0	7	2	26	0	0	0	0	0	12	2	2	0	3	0	0	0	0	11	0
**A368**	0	0	0	0	0	0	2	0	1	0	17	1	0	0	0	0	1	0	0	4	0	0	0	0	1	6
**A405**	1	1	2	1	0	1	0	1	1	0	1	232	1	0	0	1	1	2	0	5	1	0	2	0	0	1
**A418**	2	2	1	1	0	2	1	5	5	0	0	1	147	4	94	7	2	4	5	5	2	0	6	5	0	3
**A420**	1	1	0	0	0	2	0	2	0	0	0	0	4	61	3	1	0	2	44	3	1	0	1	3	2	1
**A434**	0	2	0	0	1	2	0	3	4	0	0	0	94	3	526	2	1	2	1	3	5	0	3	2	1	0
**A440**	10	13	6	6	1	3	3	31	21	12	0	1	7	1	2	129	5	7	3	23	3	0	7	4	12	3
**A476**	3	13	1	0	0	250	2	8	6	2	1	1	2	0	1	5	352	7	0	38	0	0	55	4	2	0
**A482**	4	7	2	6	1	1	0	5	7	2	0	2	4	2	2	7	7	79	0	12	4	0	6	5	5	2
**A608**	0	0	0	1	0	2	0	1	0	0	0	0	5	44	1	3	0	0	158	2	1	0	1	1	1	1
**A622**	11	13	7	11	1	33	3	18	24	3	4	5	5	3	3	23	38	12	2	204	3	1	10	4	8	6
**A623**	1	3	1	4	1	0	1	4	3	0	0	1	2	1	5	3	0	4	1	3	19	0	3	1	2	1
**A636**	0	0	0	0	0	0	0	0	0	0	0	0	0	0	0	0	0	0	0	1	0	37	0	0	0	0
**A670**	5	16	3	3	1	1	1	14	8	0	0	2	6	1	3	7	55	6	1	10	3	0	308	5	1	2
**A792**	2	3	0	1	0	2	2	5	2	0	0	0	5	3	2	4	4	5	1	4	1	0	5	128	1	1
**A809**	4	4	0	0	1	1	0	8	3	11	1	0	0	2	1	12	2	5	1	8	2	0	1	1	198	1
**A832**	3	2	3	1	2	1	0	3	4	0	6	1	3	1	0	3	0	2	1	6	1	0	2	1	1	23

Supplementary Table 3 Matrix of the shared OTUs among samples in the "no myco" dataset used for Fig. 6C.

	A032	A138	A172	A194	A227	A229	A243	A280	A360	A361	A368	A405	A418	A420	A434	A440	A476	A482	A608	A622	A623	A636	A670	A792	A809	A832
**A032**	19	6	6	6	0	0	0	8	9	1	0	1	2	0	0	10	2	3	0	9	1	0	5	1	3	1
**A138**	6	131	5	3	1	0	4	18	10	2	0	1	2	0	2	12	13	6	0	12	3	0	16	2	3	1
**A172**	6	5	172	5	0	0	0	8	6	0	0	2	1	0	0	6	1	2	0	6	1	0	3	0	0	1
**A194**	6	3	5	109	0	0	0	5	8	0	0	1	1	0	0	6	0	5	1	10	3	0	3	0	0	1
**A227**	0	1	0	0	9	0	0	0	1	0	0	0	0	0	1	1	0	1	0	1	1	0	1	0	1	1
**A229**	0	0	0	0	0	64	0	2	3	2	0	1	2	1	2	3	51	0	1	3	0	0	1	1	1	0
**A243**	0	4	0	0	0	0	18	3	0	0	0	0	0	0	0	2	1	0	0	1	1	0	1	2	0	0
**A280**	8	18	8	5	0	2	3	87	14	7	0	1	4	1	3	30	8	4	1	16	4	0	14	4	7	1
**A360**	9	10	6	8	1	3	0	14	77	2	0	1	4	0	4	21	4	6	0	21	3	0	8	2	3	4
**A361**	1	2	0	0	0	2	0	7	2	25	0	0	0	0	0	12	2	2	0	3	0	0	0	0	11	0
**A368**	0	0	0	0	0	0	0	0	0	0	2	1	0	0	0	0	1	0	0	1	0	0	0	0	0	1
**A405**	1	1	2	1	0	1	0	1	1	0	1	228	1	0	0	1	1	2	0	5	1	0	2	0	0	1
**A418**	2	2	1	1	0	2	0	4	4	0	0	1	80	3	60	4	2	3	1	2	1	0	4	4	0	1
**A420**	0	0	0	0	0	1	0	1	0	0	0	0	3	51	3	0	0	0	38	1	0	0	0	1	0	0
**A434**	0	2	0	0	1	2	0	3	4	0	0	0	60	3	491	2	1	2	1	3	5	0	3	2	1	0
**A440**	10	12	6	6	1	3	2	30	21	12	0	1	4	0	2	114	5	6	2	21	3	0	6	3	12	2
**A476**	2	13	1	0	0	51	1	8	4	2	1	1	2	0	1	5	132	6	0	14	0	0	55	3	2	0
**A482**	3	6	2	5	1	0	0	4	6	2	0	2	3	0	2	6	6	67	0	9	4	0	4	3	4	1
**A608**	0	0	0	1	0	1	0	1	0	0	0	0	1	38	1	2	0	0	146	1	0	0	0	1	0	0
**A622**	9	12	6	10	1	3	1	16	21	3	1	5	2	1	3	21	14	9	1	143	2	1	8	2	6	1
**A623**	1	3	1	3	1	0	1	4	3	0	0	1	1	0	5	3	0	4	0	2	13	0	3	0	1	1
**A636**	0	0	0	0	0	0	0	0	0	0	0	0	0	0	0	0	0	0	0	1	0	37	0	0	0	0
**A670**	5	16	3	3	1	1	1	14	8	0	0	2	4	0	3	6	55	4	0	8	3	0	277	4	1	1
**A792**	1	2	0	0	0	1	2	4	2	0	0	0	4	1	2	3	3	3	1	2	0	0	4	27	0	0
**A809**	3	3	0	0	1	1	0	7	3	11	0	0	0	0	1	12	2	4	0	6	1	0	1	0	190	0
**A832**	1	1	1	1	1	0	0	1	4	0	1	1	1	0	0	2	0	1	0	1	1	0	1	0	0	9

Supplementary Table 4 Matrix of the shared OTUs among samples of *Lecanora* spp. in the "no myco" dataset used for Fig. 6D.

	A227	A243	A360	A368	A418	A434	A482	A670	A832
**A227**	9	0	1	0	0	1	1	1	1
**A243**	0	18	0	0	0	0	0	1	0
**A360**	1	0	77	0	4	4	6	8	4
**A368**	0	0	0	2	0	0	0	0	1
**A418**	0	0	4	0	80	60	3	4	1
**A434**	1	0	4	0	60	491	2	3	0
**A482**	1	0	6	0	3	2	67	4	1
**A670**	1	1	8	0	4	3	4	277	1
**A832**	1	0	4	1	1	0	1	1	9

Supplementary Table 5 Matrix of the shared OTUs among samples of *Rhizocarpon geographicum* in the "no myco" dataset used for Fig. 6E.

	A172	A194	A405
**A172**	172	5	2
**A194**	5	109	1
**A405**	2	1	228

Supplementary Table 6 Matrix of the shared OTUs among samples of *Tephromela atra* in the "no myco" dataset used for Fig. 6F.

	A280	A361	A440	A809
**A280**	87	7	30	7
**A361**	7	25	12	11
**A440**	30	12	114	12
**A809**	7	11	12	190

Supplementary Table 7 Matrix of the shared OTUs belonging to Capnodiales in the complete dataset used for Fig. 7A.

	A280	A360	A440	A622	A809
**A280**	7	1	2	1	0
**A360**	1	1	0	1	0
**A440**	2	0	2	0	0
**A622**	1	1	0	9	0
**A809**	0	0	0	0	21

Supplementary Table 8 Matrix of the shared OTUs belonging to Chaetothyriales in the complete dataset used for Fig. 7B.

	**A032**	**A138**	**A172**	**A243**	**A280**	**A360**	**A368**	**A405**	**A418**	**A434**	**A440**	**A476**	**A482**	**A622**	**A623**	**A670**	**A792**
**A032**	1	1	1	0	1	1	0	0	0	0	1	1	0	0	0	1	0
**A138**	1	14	2	1	6	1	0	0	0	0	3	5	0	2	0	5	1
**A172**	1	2	5	0	4	1	0	0	0	0	2	1	0	0	0	1	0
**A243**	0	1	0	2	1	0	0	0	0	0	0	1	0	1	0	1	1
**A280**	1	6	4	1	25	2	0	0	0	0	4	2	1	1	2	3	1
**A360**	1	1	1	0	2	3	0	0	0	0	1	1	0	0	0	1	0
**A368**	0	0	0	0	0	0	1	1	0	0	0	1	0	1	0	0	0
**A405**	0	0	0	0	0	0	1	1	0	0	0	1	0	1	0	0	0
**A418**	0	0	0	0	0	0	0	0	2	1	0	0	0	0	0	0	0
**A434**	0	0	0	0	0	0	0	0	1	11	0	0	0	0	0	0	0
**A440**	1	3	2	0	4	1	0	0	0	0	9	1	0	0	0	1	0
**A476**	1	5	1	1	2	1	1	1	0	0	1	28	0	4	0	26	1
**A482**	0	0	0	0	1	0	0	0	0	0	0	0	5	0	1	0	0
**A622**	0	2	0	1	1	0	1	1	0	0	0	4	0	4	0	3	1
**A623**	0	0	0	0	2	0	0	0	0	0	0	0	1	0	2	0	0
**A670**	1	5	1	1	3	1	0	0	0	0	1	26	0	3	0	36	1
**A792**	0	1	0	1	1	0	0	0	0	0	0	1	0	1	0	1	1

Supplementary Table 9 Matrix of the shared OTUs belonging to Tremellales in the complete dataset used for Fig. 7C.

	**A138**	**A280**	**A360**	**A418**	**A434**	**A440**	**A476**	**A482**	**A608**	**A622**	**A623**	**A670**	**A792**
**A138**	1	1	0	0	0	1	0	0	0	0	0	0	0
**A280**	1	3	1	0	0	2	0	0	0	0	1	1	0
**A360**	0	1	4	2	1	1	1	1	0	2	1	2	1
**A418**	0	0	2	4	1	0	2	2	0	1	0	1	2
**A434**	0	0	1	1	1	0	1	1	0	1	0	1	1
**A440**	1	2	1	0	0	2	0	0	0	0	1	1	0
**A476**	0	0	1	2	1	0	2	2	0	1	0	1	2
**A482**	0	0	1	2	1	0	2	3	0	1	0	1	3
**A608**	0	0	0	0	0	0	0	0	0	0	0	0	0
**A622**	0	0	2	1	1	0	1	1	0	3	0	1	1
**A623**	0	1	1	0	0	1	0	0	0	0	1	1	0
**A670**	0	1	2	1	1	1	1	1	0	1	1	2	1
**A792**	0	0	1	2	1	0	2	3	0	1	0	1	4

Supplementary Table 10 Number of OTUs and corresponding reads of two cultured fungi (A930 and 1022, Muggia *et al.* 2016), found in the complete dataset. The reads found in samples with the same lichen-lichenicolous fungus combination from which culture was isolated are highlighted in bold.

	**Cultured fungus**	
	**A930**	**A1022**
**Sample**		
A032	OTU47 (116)	-
A138	OTU47 (11)	-
A172	OTU47 (57)	-
A194	OTU47 (426)	-
A227	-	-
A229	-	-
A243	-	-
A280	OTU47 (37)	-
A360	OTU47 (246)	OTU9873 (37), OTU3048 (1), OTU10383 (1)
A361	-	-
A368	-	-
A405	OTU47 (3)	-
A418	OTU47 (2)	-
A420	-	-
A434	-	-
A440	**OTU47 (131)**	-
A476	-	-
A482	OTU47 (12)	-
A608	-	-
A622	OTU47 (572), OTU8759 (2)	-
A623	OTU47 (1)	-
A636	-	-
A670	OTU47 (1)	-
A792	-	-
A809	-	-
A832	OTU47 (1)	OTU9873 (57), OTU3048 (1), OTU10383 (1)
